# Effects of land cover and protected areas on flying insect diversity

**DOI:** 10.1111/cobi.14425

**Published:** 2024-12-04

**Authors:** James S. Sinclair, Dominik Buchner, Mark O. Gessner, Jörg Müller, Steffen U. Pauls, Stefan Stoll, Ellen A. R. Welti, Claus Bässler, Jörn Buse, Frank Dziock, Julian Enss, Thomas Hörren, Robert Künast, Yuanheng Li, Andreas Marten, Carsten Morkel, Ronny Richter, Sebastian Seibold, Martin Sorg, Sönke Twietmeyer, Dirk Weis, Wolfgang Weisser, Benedikt Wiggering, Martin Wilmking, Gerhard Zotz, Mark Frenzel, Florian Leese, Peter Haase

**Affiliations:** ^1^ Department of River Ecology and Conservation Senckenberg Research Institute and Natural History Museum Frankfurt Gelnhausen Germany; ^2^ Aquatic Ecosystem Research University of Duisburg‐Essen Essen Germany; ^3^ Department of Plankton and Microbial Ecology Leibniz Institute of Freshwater Ecology & Inland Fisheries (IGB) Stechlin Germany; ^4^ Department of Ecology Berlin Institute of Technology (TU Berlin) Berlin Germany; ^5^ Field Station Fabrikschleichach, Department of Animal Ecology and Tropical Biology Biocenter University of Würzburg Rauhenebrach Germany; ^6^ Nationalpark Bavarian Forest Grafenau Germany; ^7^ Terrestrial Zoology Department Senckenberg Research Institute and Nature Museum Frankfurt Frankfurt am Main Germany; ^8^ Institute of Insect Biotechnology Justus‐Liebig‐University Gießen Giessen Germany; ^9^ LOEWE Centre for Translational Biodiversity Genomics Frankfurt am Main Germany; ^10^ Environmental Campus Birkenfeld University of Applied Sciences Trier Hoppstädten‐Weiersbach Germany; ^11^ Faculty of Biology University of Duisburg‐Essen Essen Germany; ^12^ Conservation Ecology Center Smithsonian's National Zoo and Conservation Biology Institute Front Royal Virginia USA; ^13^ Ecology of Fungi University Bayreuth Bayreuth Germany; ^14^ Department for Ecological Monitoring, Research and Species Protection Black Forest National Park Freudenstadt Germany; ^15^ Faculty of Agriculture/Environment/Chemistry University of Applied Sciences HTW Dresden Dresden Germany; ^16^ Aquatic Ecology, Faculty of Biology University of Duisburg‐Essen Essen Germany; ^17^ Entomological Society Krefeld (EVK) Krefeld Germany; ^18^ Terrestrial Ecology Research Group, Department of Life Science Systems, School of Life Sciences Technical University of Munich Freising Germany; ^19^ Harz National Park Wernigerode Germany; ^20^ Kellerwald‐Edersee National Park Bad Wildungen Germany; ^21^ German Centre for Integrative Biodiversity Research (iDiv) Halle‐Jena‐Leipzig Leipzig Germany; ^22^ Systematic Botany and Functional Biodiversity, Institute for Biology Leipzig University Leipzig Germany; ^23^ Berchtesgaden National Park Berchtesgaden Germany; ^24^ Ecosystem Dynamics and Forest Management, Department of Life Science Systems, School of Life Sciences Technical University of Munich Freising Germany; ^25^ Forest Zoology TUD Dresden University of Technology Tharandt Germany; ^26^ Department of Research and Documentation Eifel National Park Schleiden Germany; ^27^ Biosphärenreservat Oberlausitzer Heide‐ und Teichlandschaft Malschwitz Germany; ^28^ Lower Saxon Wadden Sea National Park Authority Wilhelmshaven Germany; ^29^ Institute for Botany and Landscape Ecology University Greifswald Greifswald Germany; ^30^ Institute for Biology and Environmental Sciences Carl von Ossietzky Universität Oldenburg Oldenburg Germany; ^31^ Department of Community Ecology Helmholtz Centre for Environmental Research UFZ Halle Germany; ^32^ Centre for Water and Environmental Research (ZWU) University of Duisburg‐Essen Essen Germany

**Keywords:** biodiversity, biomass, climate change, insect, land cover, metabarcoding, pollinator, protected area, área protegida, biodiversidad, biomasa, cambio climático, cobertura de suelo, insecto, metacodificación de barras, polinizador

## Abstract

Widespread insect losses are a critical global problem. Mitigating this problem requires identifying the principal drivers across different taxa and determining which insects are covered by protected areas. However, doing so is hindered by missing information on most species owing to extremely high insect diversity and difficulties in morphological identification. To address this knowledge gap, we used one of the most comprehensive insect DNA metabarcoding data sets assembled (encompassing 31,846 flying insect species) in which data were collected from a network of 75 Malaise traps distributed across Germany. Collection sites encompass gradients of land cover, weather, and climate, along with differences in site protection status, which allowed us to gain broader insights into how insects respond to these factors. We examined changes in total insect biomass, species richness, temporal turnover, and shifts in the composition of taxa, key functional groups (pollinators, threatened species, and invasive species), and feeding traits. Lower insect biomass generally equated to lower richness of all insects and higher temporal turnover, suggesting that biomass loss translates to biodiversity loss and less stable communities. Spatial variability in insect biomass and composition was primarily driven by land cover, rather than weather or climate change. As vegetation and land‐cover heterogeneity increased, insect biomass increased by 50% in 2019 and 56% in 2020 and total species richness by 58% and 33%, respectively. Similarly, areas with low‐vegetation habitats exhibited the highest richness of key taxa, including pollinators and threatened species, and the widest variety of feeding traits. However, these habitats tended to be less protected despite their higher diversity. Our results highlight the value of heterogeneous low vegetation for promoting overall insect biomass and diversity and that better protection of insects requires improved protection and management of unforested areas, where many biodiversity hotspots and key taxa occur.

## Abstract (es)

Efectos de la cobertura del suelo y las áreas protegidas sobre la diversidad de insectos voladores

**Resumen:**

La pérdida generalizada de insectos es un problema mundial crítico. Para mitigarlo, es necesario identificar los principales factores que lo provocan en los distintos taxones y determinar qué insectos están cubiertos por áreas protegidas. Sin embargo, la falta de información sobre la mayoría de las especies, debido a la gran diversidad de insectos y a las dificultades de identificación morfológica, dificulta esta tarea. Para cerrar este vacío de conocimiento utilizamos uno de los conjuntos más completos de datos de metacodificación de barras de ADN de insectos reunidos (que abarca 31,846 especies de insectos voladores) en el que se recogieron datos de una red de 75 trampas Malaise distribuidas por toda Alemania. Los puntos de colecta abarcaron gradientes de cobertura del suelo, tiempo, clima y diferencias en el estado de protección del lugar, lo que nos permitió obtener una visión más amplia de cómo responden los insectos a estos factores. Analizamos los cambios en la biomasa total de insectos, la riqueza de especies, la rotación temporal y los cambios en la composición de taxones, grupos funcionales clave (polinizadores, especies amenazadas y especies invasoras) y rasgos de alimentación. Una menor biomasa de insectos equivale en general a una menor riqueza de todos los insectos y a una mayor rotación temporal, lo que sugiere que la pérdida de biomasa se traduce en pérdida de biodiversidad y en comunidades menos estables. La variabilidad espacial de la biomasa y la composición de los insectos se debió principalmente a la cubierta terrestre, más que al tiempo o al cambio climático. A medida que aumentaba la heterogeneidad de la vegetación y la cubierta terrestre, la biomasa de insectos aumentó un 50% en 2019 y un 56% en 2020, y la riqueza total de especies un 58% y un 33%, respectivamente. Del mismo modo, las zonas con hábitats de vegetación baja mostraron la mayor riqueza de taxones clave, incluidos los polinizadores y las especies amenazadas, y la mayor variedad de rasgos de alimentación. Sin embargo, estos hábitats tendían a estar menos protegidos a pesar de su mayor diversidad. Nuestros resultados ponen de relieve el valor de la vegetación heterogénea baja para promover la biomasa y la diversidad de insectos en general y que una mejor protección de los insectos requiere una mejor protección y gestión de las zonas no arboladas, donde se encuentran muchos puntos calientes de biodiversidad y taxones clave.

## INTRODUCTION

Reports of widespread insect losses (Conrad et al., 2006; Outhwaite et al., 2022; Raven & Wagner, 2021; Soroye et al., 2020) and associated declines in key ecosystem functions (e.g., pollination and pest control; Cardoso et al., 2020; Zhou et al., 2023) have raised global awareness of the need for better insect conservation. However, most research on insect declines has been limited to specific taxonomic or functional groups, particularly bees and butterflies (e.g., Powney et al., 2019; Soroye et al., 2020; Warren et al., 2021), or to single metrics that summarize insect communities, such as overall biomass (e.g., Hallmann et al., 2017; Lister & Garcia, 2018; Müller et al., 2023). This narrow focus raises questions about whether reported declines are only occurring in examined metrics or taxonomic groups (Saunders et al., 2020) or whether they are indicative of broader trends, such as declining biomass indicating a similar degree of species loss (Hallmann et al., 2021; van Klink et al., 2023). Additionally, high variability across studies in their methods, focal taxa, and community metrics makes it difficult to generalize findings on the drivers of insect declines. For example, changes in land cover, weather, and climate can affect community structure, but taxa vary in their responses to each driver (Ganuza et al., 2022; Outhwaite et al., 2020). The identity of the examined taxonomic group may therefore explain why some researchers find impacts of just land cover or just meteorological factors (e.g., Engelhardt et al., 2022; Englmeier et al., 2023), whereas others find both to be important (e.g., Neff et al., 2022; Oliver et al., 2016). Studies that simultaneously examine a wider variety of taxa and multiple community metrics are therefore needed to better determine how insects are responding to environmental change.

A broader perspective of insect communities is also needed to inform conservation actions aimed at protecting insect diversity and controlling invasive species. Protected areas, such as nature reserves and national parks, are typically designated to protect wildlife, but the location and design of these areas rarely consider insects (Chowdhury et al., 2023). Consequently, little is known about which insects are covered by current protected areas and whether these areas are protecting vulnerable native species, threatened species, and key functional groups (e.g., pollinators and dung beetles). Native and threatened insects are also at risk of further decline owing to the ongoing spread of invasive insects (Fortuna et al., 2022), which can be sources of costly ecosystem disservices, such as pests or disease vectors (Bradshaw et al., 2016). Changes in and drivers of invasive insects can be missed if researchers examine only certain taxonomic subsets or community metrics.

At present, obtaining a more comprehensive perspective of insect communities is hindered by insufficient knowledge of species diversity and distribution. Insects are by far the most diverse animal group on Earth, comprising millions of known and as yet unknown and cryptic species (Li & Wiens, 2022; Mora et al., 2011). This extremely high diversity partly explains why many studies have a limited taxonomic scope because extensive expertise, time, and cost can be required to identify all insects, particularly across large spatiotemporal scales that can encompass thousands of species (e.g., Karlsson et al., 2020; Svenningsen et al., 2021). Genetic identification methods, such as DNA metabarcoding (hereafter metabarcoding), offer a promising solution to this problem. Metabarcoding involves sequencing a mixed pool of DNA and identifying taxa through comparison with sequences in DNA reference libraries. This process avoids the need to examine specimens individually, allowing for rapid, cost‐effective, and high‐resolution identification that can detect thousands of insect species within a few weeks (Buchner et al., 2024). Additionally, metabarcoding can help detect unknown taxa by, for example, defining putative species via operational taxonomic units (OTUs). Metabarcoding can also identify species that are difficult to distinguish morphologically and can more effectively track the presence of invasive insects (Piper et al., 2019). Consequently, genetic identification methods can expand the scope of insect community analyses to more taxa at broader spatial scales. These methods have thus become key tools for monitoring global insect biodiversity (e.g., Seymour et al., 2024; Srivathsan et al., 2023) and revealing the principal processes structuring communities (e.g., Ganuza et al., 2022; Uhler et al., 2021).

**FIGURE 1:**
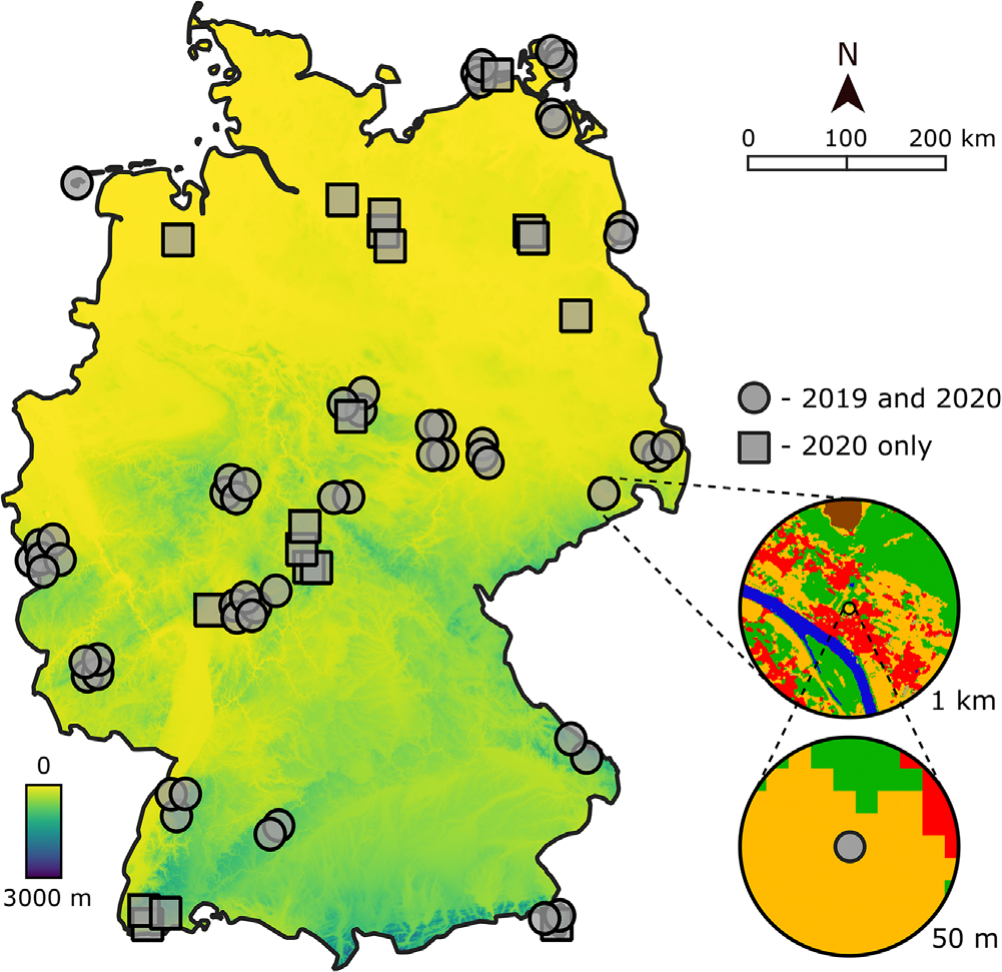
Locations of 75 insect sampling sites (Malaise traps) across Germany. Sites spanned an elevation gradient of 1–1400 m. Insets illustrate differences in how we measured fine‐scale (50‐m radius around each trap) versus broadscale (1‐km radius) land cover (green, forest; orange, low vegetation; red, urban; brown, agricultural; bare soil, not shown). Some trap positions are shifted slightly to improve visualization. Site coordinates and environmental characteristics in Appendix S1.

We took advantage of recent advances in metabarcoding technology (detailed in Buchner et al. [2024]) to analyze patterns and drivers of flying insect communities across Germany. Insects were collected during 2019 and 2020 from a network of 75 monitoring sites (Figure 1). The resulting data set on 31,846 species provides one of the most comprehensive, broadscale assessments of insect communities. We asked 3 primary questions: Do seasonal and site‐level changes in insect biomass translate to equivalent changes in other community metrics, specifically species richness and a measure of temporal community change within sites (hereafter temporal turnover) (Q1); how do different community metrics and insect taxa respond to spatial land cover, weather, and climatic gradients (Q2); and how do these factors vary between protected versus unprotected areas (Q3)?

We addressed the latter 2 questions by determining insect biomass, diversity, taxonomic composition, and feeding trait composition. We also examined responses in community subsets of key importance to insect conservation, specifically threatened species, pollinators, and invasive species. We hypothesized that both insect biomass and species richness are lower in sites surrounded by more anthropogenic land cover (e.g., urban and agricultural areas) and under warmer and drier meteorological conditions, given the known negative effects of these stressors on insects (Cardoso et al., 2020; Müller et al., 2023; Outhwaite et al., 2022; Raven & Wagner, 2021). Temporal turnover may also be higher because lower diversity communities can be less temporally stable (e.g., Senapathi et al., 2021). Conversely, we expected higher biomass, higher richness, and lower temporal turnover in protected areas that restrict human activity and land modification (Jones et al., 2018) because they help maintain the environmental conditions certain insects require, such as low temperatures (Thomas & Gillingham, 2015).

## METHODS

### Study sites

Our insect monitoring network comprised 56 sites across Germany during 2019 plus 19 sites added in 2020 to expand spatial coverage (Figure 1). These 75 sites were either already being monitored to address other insect research questions, such as effects of agriculture, or selected to expand the network into special habitat types, such as protected areas (further site information is available from https://www.ufz.de/lter‐d/index.php?de=46285 under the “LTER‐D Sites” heading). Consequently, the sites captured a diverse range of forested, unforested, urban, and agricultural land‐cover types, including protected and unprotected areas and gradients in weather and climate change (detailed below).

### Insect sampling

Townes‐type Malaise traps with black roofs and 1.16‐m^2^ openings on each side were set up at each site (coordinates in Appendix S1). Traps were primarily placed in open areas (typically agricultural fields or grasslands), adjacent to forest edges or hedgerows, or in forest clearings. Eleven traps were placed under forest canopies (Appendix S2). Trap openings usually faced east‐west. The highest trap section, where trapped insects are funneled and collected, was aligned according to the conditions on site, often toward the sun in the south, southwest, or southeast in Germany. Traps were typically deployed from April through October in 2019 and 2020, although some traps were deployed earlier or later depending on logistics and weather. All trapped insects were generally collected every 14 days, and new sample bottles were set up after each collection. Shorter and longer sampling periods, ranging from 7 to 29 days, were occasionally necessary owing to logistical constraints. A total of 856 samples were collected across sites in 2019, and 1084 were collected in 2020. Of these samples, 4 provided no metabarcoding results and 121 were withheld for use in other analyses; thus, these provided biomass data but no community information.

### Biomass and metabarcoding

All captured insects were preserved in 80% denatured ethanol (1% methyl ethyl ketone) and transported to the lab to determine wet biomass and species identity via metabarcoding. To measure biomass, samples were sieved (0.8‐mm mesh) following the procedure in Hallmann et al. (2017). Filtered samples were then weighed to the nearest 0.01 g.

Metabarcoding methods are fully detailed in Buchner et al. (2024). In brief, insect DNA was amplified using the fwh2 forward and fwhR2n reverse primers targeting a 205‐bp fragment (Vamos et al., 2017). Each sample had 2 technical replicates, and each 96‐well plate included 12 negative controls, which were clean after replicate merging. Sequencing was performed using the HiSeq X platform (Macrogen Europe, 2 × 150 cycles) with at least 1 million reads per sample. Reads were processed with the APSCALE pipeline, and taxonomy was assigned via the Barcode of Life Database (BOLD) with BOLDigger. OTUs could not always be assigned to species names because of incomplete reference data or conflicting matches in the databases. Therefore, OTUs were divided into 2 groups. The first group included OTUs that could be unambiguously matched to a barcode with a species name, encompassing 10,803 species that we termed *validated species*. The second group also included OTUs that could only be resolved to genus or family level. These OTUs were used to estimate the likely species richness of each insect family (Appendix S3), which provided an additional 21,043 species that we termed *plausible species*.

### Insect community metrics

We quantified total insect biomass (grams), species richness, and temporal turnover for each sample collected during 2019 and 2020. Temporal turnover was calculated as a shift in species presence or absence from one sample to the next successive sample from the same site and year based on Jaccard's index, which ranges from 0 (no compositional change) to 1.

We quantified insect community composition for each sample based on broad‐ and fine‐scale taxonomic factors, including key functional groups (threatened species, pollinators, and invasive species) and feeding traits. We did so to determine whether responses differed between taxonomic levels (i.e., insect orders vs. families) and based on species or species’ traits. For the broad scale, we quantified the richness of the 5 most diverse insect orders in our data set: Coleoptera, Diptera, Hemiptera, Hymenoptera, and Lepidoptera. For the fine scale, we quantified richness for 86 insect groups in these orders: 125 focal families (out of 359 total) and >90% of all species (Appendix S4). We focused on this subset of families because they represented the most species and to produce interpretable ordination plots that would have been cluttered if we had included all 359 families. Threatened species were assigned using a country‐level red list of species considered endangered (categories 1, 2, 3, R, and G) in Germany (Hallmann et al., 2022). For pollinators, we calculated richness of bees, butterflies, and hoverflies (Appendix S4). Invasive species were determined based on species considered non‐native and invasive or potentially invasive in Germany (sources in Appendix S5). Feeding trait composition was determined using larval trait values from Hörren et al. (2022), which were available for 89% of our identified families.

### Land cover

To determine how insects responded to changes in land cover (Q2), we extracted land‐cover data for each site in 2019 and 2020 from 10‐m resolution raster maps of agricultural, bare soil, forest, low vegetation, and urban land‐cover types in Germany derived from Sentinel‐2 satellite imagery (Riembauer et al., 2021). We extracted the proportions of each land‐cover type in fine‐scale (50‐m radius around the collection site) and broadscale (1‐km radius) areas to capture the potential influence of land cover in the immediate surroundings on insects with limited mobility and of land cover in a larger area on more mobile taxa (e.g., Riva et al., 2023), respectively. We considered including land cover within 500 m and 5 km, but these cover values were generally correlated to the 1‐km values (mean *r* of 0.91 and 0.68, respectively). In addition to the 5 land‐cover types, we included a measure of land‐cover heterogeneity, which can also influence insect diversity (Wang et al., 2019). We calculated land‐cover heterogeneity for each site and radius with the Shannon diversity index (*H*) (range 0, 100% cover of a single land‐cover type, to 1.6, equal distribution of all 5 land‐cover types).

### Weather and climate change

To determine insect responses to weather and climate (Q2), we obtained data to represent short‐term differences in weather and long‐term climate change. We collated gridded data on daily mean temperature (degrees Celsius, 5 × 5‐km resolution), daily total precipitation (millimeters, 1 × 1‐km resolution), and daily mean percent relative humidity (5 × 5‐km resolution) from the German Weather Service (https://opendata.dwd.de), which is interpolated from stations across Central Europe. We used these data to calculate weather averaged for each site and year across all 14‐day sampling periods (maps of 2020 values in Appendix S6).

We included climate anomalies to represent climate change (Table 2), which we calculated as the difference between the weather values for each site and year and the same periods averaged across the previous 30 years. Sampled insects can also be influenced by the degree of climate change in previous years, which can affect their development (Müller et al., 2023). To account for this, we included the difference in temperature, precipitation, and humidity for each site during April in the year before sampling compared with Aprils from the previous 30 years.

### Protected areas

To consider differences among insects based on site protection status (Q3), we extracted protection data from Protected Planet (https://www.protectedplanet.net) and Palliwoda et al. (2021). We included 4 types of protected areas in Germany that potentially affect terrestrial insects: national parks, nature reserves, biosphere reserves, and landscape protection areas. We grouped sites in national parks, nature reserves, and the inner 2 zones of biosphere reserves into a single more‐protected category. These areas all restrict land conversion for anthropogenic purposes and thus experience reduced anthropogenic pressures (Jones et al., 2018; Palliwoda et al., 2021). We grouped sites in landscape protection areas and the outer transition zone of biosphere reserves into a less‐protected category because these protection areas allow for more extensive human activity. All other sites were considered unprotected.

### Seasonal changes

As part of addressing Q1, we first compared seasonal patterns in insect biomass with species richness and temporal turnover to determine whether broad trends in biomass reflected trends in richness and turnover. We related these metrics to the final Julian day in each exposure period for each site with generalized additive mixed models (GAMMs) performed with the mgcv package (Wood, 2017) in R (R Core Team, 2023). All models included a smoothed predictor for Julian day, which varied by year, and used thin‐plate regression splines with a basis dimension of *k* = 5. We tested the need for higher basis dimensions and cyclic smoothers that might better fit seasonal data, but these changes did not alter the broad trends. Models also included a fixed categorical term for year, a random term for each site to control for repeated measures, and a first‐order autoregressive structure to control for temporal autocorrelation. We tested for positive spatial autocorrelation with Moran's eigenvector maps (Dray et al., 2012) and found evidence for autocorrelation in turnover in 2019 but not 2020; thus, we did not consider the effect consistent enough to need to be controlled. Biomass was converted to a daily rate (grams per day) to account for differences in the number of days per sampling period. We controlled for these same effects on richness with an offset. Biomass and turnover were modeled using Gaussian distributions with log‐link functions, and richness for all taxa was modeled using Poisson distributions with log‐link functions.

### Site‐level relationships

We used a series of multivariate analyses to examine relationships among different community metrics (Q1) and to land cover, weather, and climate change (Q2). We first converted the seasonal data to site‐level data by averaging biomass (grams per day), the richness of all insect groups and families (species per day), temporal turnover, and trait composition for each site across only the sampling periods from late April (after Julian day 110) to late September (up to Julian day 273). We used this time window because it was the most consistent across sites, encompassing an average of 11.6 samplings per site and including 98% of all identified species.

We first used 4 separate variation partitioning analyses to determine the degree to which insects were related to the individual effects of land cover, the individual effects of weather and climate, and the covarying effects of both, such as forests tending to also be colder and wetter. The 4 response matrices for these analyses included, respectively, insect biomass and diversity (i.e., insect biomass, temporal turnover, total richness, the species richness of the 5 major insect orders, and the richness of threatened, pollinator, and invasive); group composition (i.e., richness of the previously described insect groups as a proportion of total richness), which provided an analysis of relative rather than absolute richness; family‐level composition (i.e., proportional richness of the 86 insect groups comprising 125 focal families); and feeding trait composition. All analyses were conducted with the varpart function in the vegan package in R (Oksanen et al., 2022). Two predictor matrices were used, one including the 12 land‐cover variables (6 variables at 2 spatial scales; Table 1) and one including the 9 weather or climate variables (Table 2). All response variables were converted to *z* scores by subtracting each value from its respective mean and dividing by the standard deviation. We removed highly correlated predictors based on variance inflation factors >10.

**TABLE 1:** Mean proportion of land‐cover types (agricultural, bare soil, forest, low vegetation, and urban) and a measure of the diversity of different land‐cover types (*H*) in radii of 50 m and 1 km across sites and years encompassed by the German flying insect monitoring network.

Cover type	50‐m mean (SD)	1‐km mean (SD)	50‐m range	1‐km range	50‐m %^a^	1‐km %^a^
Agricultural	0.11 (0.25)	0.13 (0.22)	0–1	0–0.96	11	7
Bare soil	0.30 (0.09)	0.02 (0.04)	0–0.56	0–0.20	1	0
Forest	0.49 (0.41)	0.57 (0.30)	0–1	0–1	44	55
Low vegetation	0.34 (0.37)	0.20 (0.16)	0–1	0–0.63	36	11
Urban	0.03 (0.01)	0.05 (0.09)	0–0.66	0–0.58	3	3
*H*	0.42 (0.35)	0.77 (0.39)	0–1.25	0.06–1.4	–	–

^a^ Percentage of sites dominated (i.e., >50% cover) by each land‐cover type out of 75 total sites.

**TABLE 2:** Mean and range of weather and climate change factors encompassed by the German flying insect monitoring network in 2019 and 2020.

Meteorological group	Variable	2019 mean (SD)	2019 range	2020 mean (SD)	2020 range
Weather^a^ current year	Temperature (°C)	15.7 (1.4)	12.2 to 17.8	15.4 (1.3)	11.5 to 18.3
	Precipitation (mm)	2.2 (0.9)	1.2 to 5.4	2.1 (1.1)	1.3 to 6.7
	Relative humidity (%)	69.9 (3.8)	62.4 to 76.5	69.1 (4.1)	60.3 to 77.8
Climate change current year^b^	Temperature (°C)	0.8 (0.2)	0.3 to 1.3	0.5 (0.4)	−0.4 to 1.9
	Precipitation (mm)	−0.3 (0.3)	−1.4 to 0.2	−0.4 (0.5)	−1.4 to 0.7
	Relative humidity	−3.8 (1.9)	−7.3 to 1.9	−4.3 (2.5)	−9.24 to 1.5
Climate change previous April^c^	Temperature (°C)	3.7 (0.8)	1.6 to 4.7	0.8 (0.4)	0.08 to 1.7
	Precipitation (mm)	−8.5 (26.1)	−66.3 to 43.7	−19.5 (9.4)	−48.8 to 4.1
	Relative humidity (%)	−2.6 (3.6)	−11.9 to 2.2	−5.0 (3.4)	−13.2 to 1.7

^a^ Weather is represented as temperature, precipitation, and humidity during the sampling periods in each year.

^b^ Differences between weather in each year and the past 30 years.

^c^ Differences between weather values in April of the previous year and values in Aprils over the previous 30 years.

In our second set of multivariate analyses, we used 4 separate redundancy analyses (RDAs) to relate the 4 community response matrices detailed above to all land cover, weather, and climate change predictors. RDAs decompose multidimensional relationships into fewer dimensions, which allowed us to visualize relationships among all the different insect metrics and taxa and all predictors in a single analysis. Additionally, stepwise permutation tests on RDAs provide statistical support for which individual predictors explain the most community variation. All RDAs were conducted with the rda function in the vegan package. We controlled for differences between sampling years by including year as a conditioning variable. We also controlled for positive spatial autocorrelation in the compositional RDAs by including 6 continuous conditioning variables of Moran's eigenvector maps calculated using a Gabriel graph connectivity scheme. This method produced predictors representing significant (*p* < 0.05) positive spatial autocorrelation in community composition among sites. We checked for the need to control elevation, latitude, and longitude, but these variables generally did not improve the models because these differences were already accounted for by the meteorological variables (see correlations in Appendix S7). The stepwise permutation tests were performed in forward and backward directions with the ordiR2step function in the vegan package. We considered important predictors those that contributed significantly (*p* < 0.05) to improving the adjusted *R* ^2^ and that contributed >1% of explained variation.

To examine differences among more‐protected, less‐protected, and unprotected sites (Q3), we compared site compositional differences in the RDAs among the 3 protected area categories with nonparametric permutational multivariate analyses of variance (PERMANOVAs). This method determines whether more‐protected, less‐protected, and unprotected sites are in significantly (*p* < 0.05) different parts of multivariate space, thus indicating differences in insect composition. We conducted 4 PERMANOVAs, one for each of the 4 RDAs. We first extracted the site locations on the first 2 RDA axes, which always captured the majority of explained variation. The site locations were then compared with a categorical variable representing the protection status of each site. These analyses were performed with the adonis2 function from the vegan package with Euclidean distance and 10,000 permutations.

Given that RDAs do not provide effect sizes, we conducted 2 additional analyses with generalized additive models (GAMs) to quantify the effect sizes of the biomass and richness relationships to the important predictors identified in the RDAs. These analyses also allowed us to test for nonlinear relationships, which RDAs cannot model. We related total biomass (grams per day) and total species richness (species per day) to smoothed terms of the land‐cover and climate predictors determined important in the stepwise permutation tests. We then determined the relative importance of these predictors with forward selection and adjusted *R* ^2^. Smoothed terms used thin‐plate regression splines and a basis dimension of *k* = 10, which we confirmed based on comparisons with the effective degrees of freedom (edf) and whether relationships changed with increased edf. We also examined differences in relationships between years by allowing the smoothed terms to vary by year. Biomass and richness were both modeled using a Gaussian distribution with an identity‐link function. Data and code needed to repeat analyses are publicly available at https://doi.org/10.6084/m9.figshare.26022244.

## RESULTS

### Validated and plausible species

All samples combined yielded 3,999,082,169 demultiplexed read pairs, averaging 1.4 million reads per sample. Sequencing yielded 50,087 total insect OTUs, 10,803 of which were assigned validated species names. We report the results for the validated species here (taxa list in Appendix S8). Results for the plausible species estimated from OTUs are provided in Appendices S3 and S9, given the greater uncertainty in the exact number of plausible species. The principal patterns were the same for both. Of the validated species, the majority were Diptera (36%), Hymenoptera (22%), Coleoptera (17%), Lepidoptera (16%), or Hemiptera (6%). All other orders each comprised about ≤1% of all species. These 5 major orders encompassed 359 different families; 51% of species belonged to just 20 families, particularly families from Hymenoptera (e.g., Ichneumonidae) and Diptera (e.g., Chironomidae, Mycetophilidae, and Sciaridae) (Appendix S9).

### Seasonal dynamics of biomass, richness, and turnover (Q1)

**FIGURE 2:**
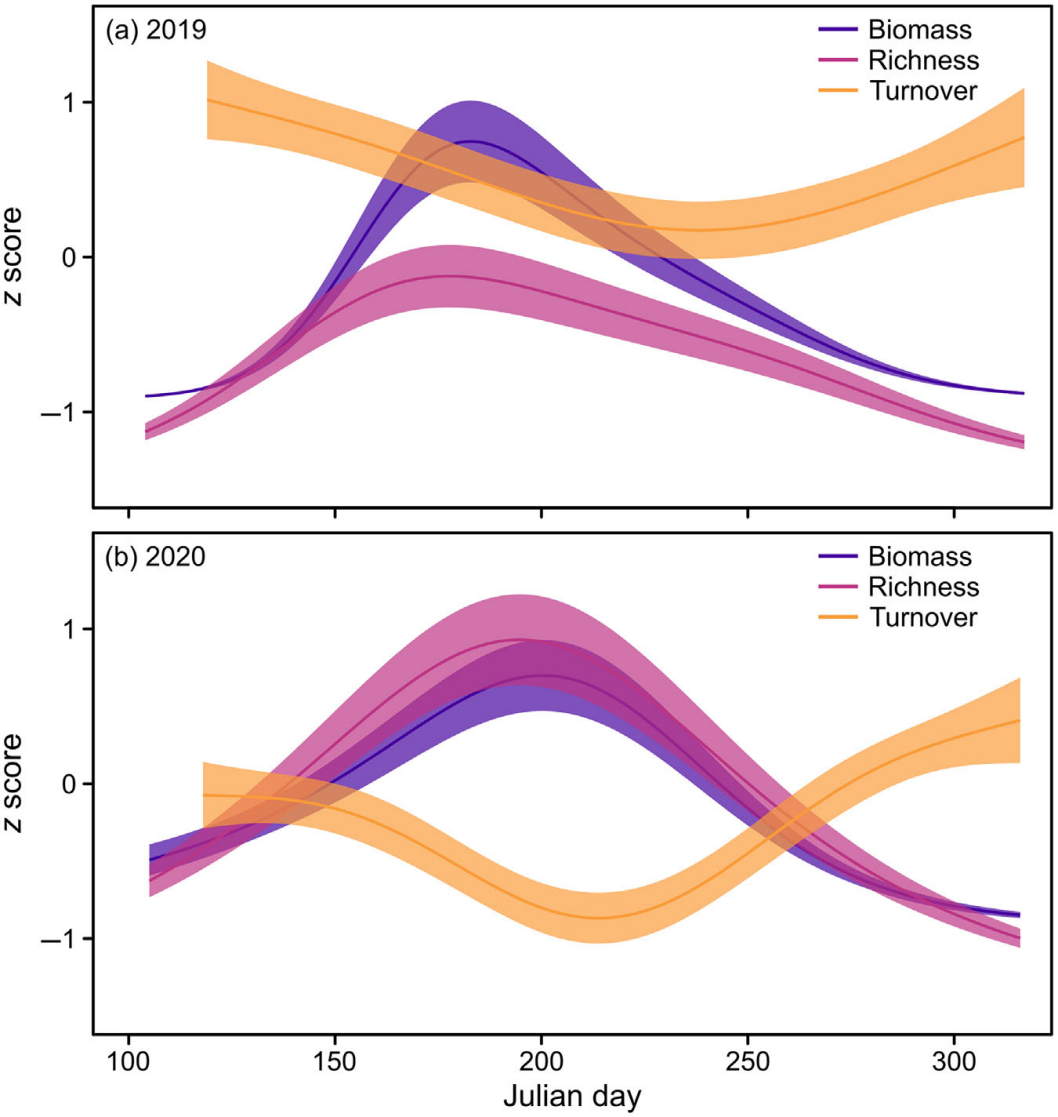
Seasonal trends in biomass (purple), total species richness (pink), and temporal turnover (orange; i.e., change in species composition in each site between successive sampling periods) of flying insects caught in Malaise traps from April through October in (a) 2019 and (b) 2020. Modeled relationships are based on generalized additive mixed model outcomes (*z* scores standardized; solid lines, best fit; shading, 95% confidence intervals).

Biomass, richness, and temporal turnover exhibited similar seasonal patterns across both years. Biomass and richness exhibited parallel concave‐down relationships, whereas temporal turnover exhibited a concave‐up relationship (Figure 2). Biomass averaged 2.0 g/day (SD 1.2) per site in 2019 and 2.3 g/day (1.2) in 2020. We also caught an average of 1229 (494) species per site in 2019 and 1700 (557) in 2020. Biomass peaked around 3.9 g/day on Julian day 183 in 2019 (early July) (*n* = 856, edf = 3.9) and at 3.8 g/day on Julian day 200 in 2020 (*n* = 1084, edf = 3.9) (Appendix S10). Total species richness mirrored changes in biomass, although richness peaked earlier and lower in 2019 (287 species on Julian day 178) (*n* = 775, edf = 3.8) than in 2020 (506 species on Julian day 195) (*n* = 1040, edf = 3.9). The species richness of the 5 major insect orders (Coleoptera, Diptera, Hemiptera, Hymenoptera, and Lepidoptera), pollinators, and threatened species generally followed similar seasonal patterns as total species richness, although invasive species tended to increase through the year (Appendix S10).

Temporal turnover was generally high throughout the year. Values were always >0.6, equating to >60% compositional change from one sampling period to the next. Turnover was lowest during August, after the period of highest biomass and richness (Figure 2), specifically, 0.71 on Julian day 238 in 2019 (*n* = 716, edf = 3.0) and 0.63 on Julian day 214 in 2020 (*n* = 945, edf = 3.8).

### Overall drivers of site‐level insect biomass, diversity, and composition (Q2)

Based on variation partitioning, land cover, weather, and climate explained 42% of the total site‐level variability in insect biomass and diversity (Appendix S11). These predictors explained 29% of differences in group composition, 25% of family‐level composition, and 36% of trait composition. The individual effect of land cover was the primary driver in all relationships and accounted for, respectively, 48%, 59%, 72%, and 69% of the total explained variation across the 4 variation partitioning analyses (Appendix S11). The individual effect of weather and climate was of less importance, explaining 26%, 31%, 24%, and 25%, respectively. The covarying effect of land cover, weather, and climate comprised the remainder (26%, 10%, 4%, and 6%, respectively).

### Individual drivers of site‐level insect biomass, diversity, and composition (Q1 & Q2)

**FIGURE 3:**
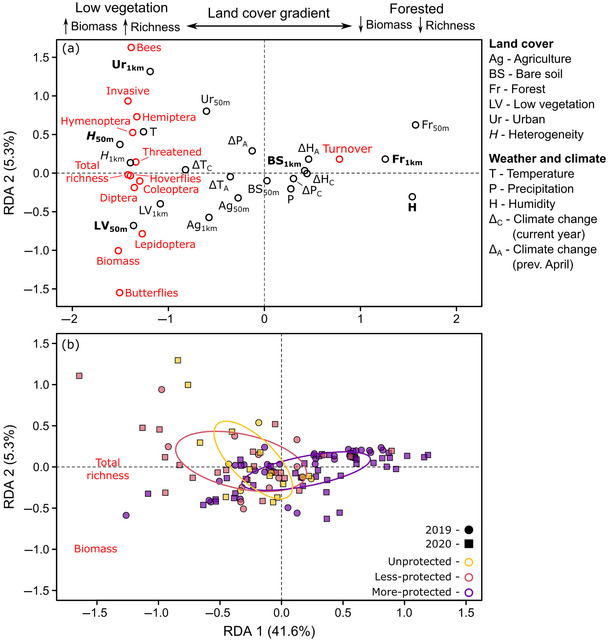
Redundancy analysis (RDA) of (a) insect biomass, temporal turnover, total species richness, and richness of different insect groups (red points) in relation to land cover, weather, and climate (black points) (50 m, fine‐scale land cover; 1 km, broadscale land cover; bold type, predictors that consistently explain the most variation based on a stepwise model selection procedure [Appendix S14]) and (b) insect community composition of each site (points) (point colors, level of site protection; ellipses, central tendency for each protection category based on standard deviations). Pollinator groups (bees, butterflies, and hoverflies) are shown separately, and their corresponding orders (Hymenoptera, Lepidoptera, and Diptera) include these groups.

Based on the insect composition and diversity RDA, sites with more low vegetation (including urban and agricultural areas) and warmer and drier conditions exhibited higher total insect biomass, higher richness of all insects, and lower temporal turnover (i.e., sites on the left side of Figure 3). In contrast, when low vegetation was scarce and conditions were colder and wetter, typically in forested areas (i.e., the right side of Figure 3), biomass and richness were lower and turnover was higher.

**FIGURE 4:**
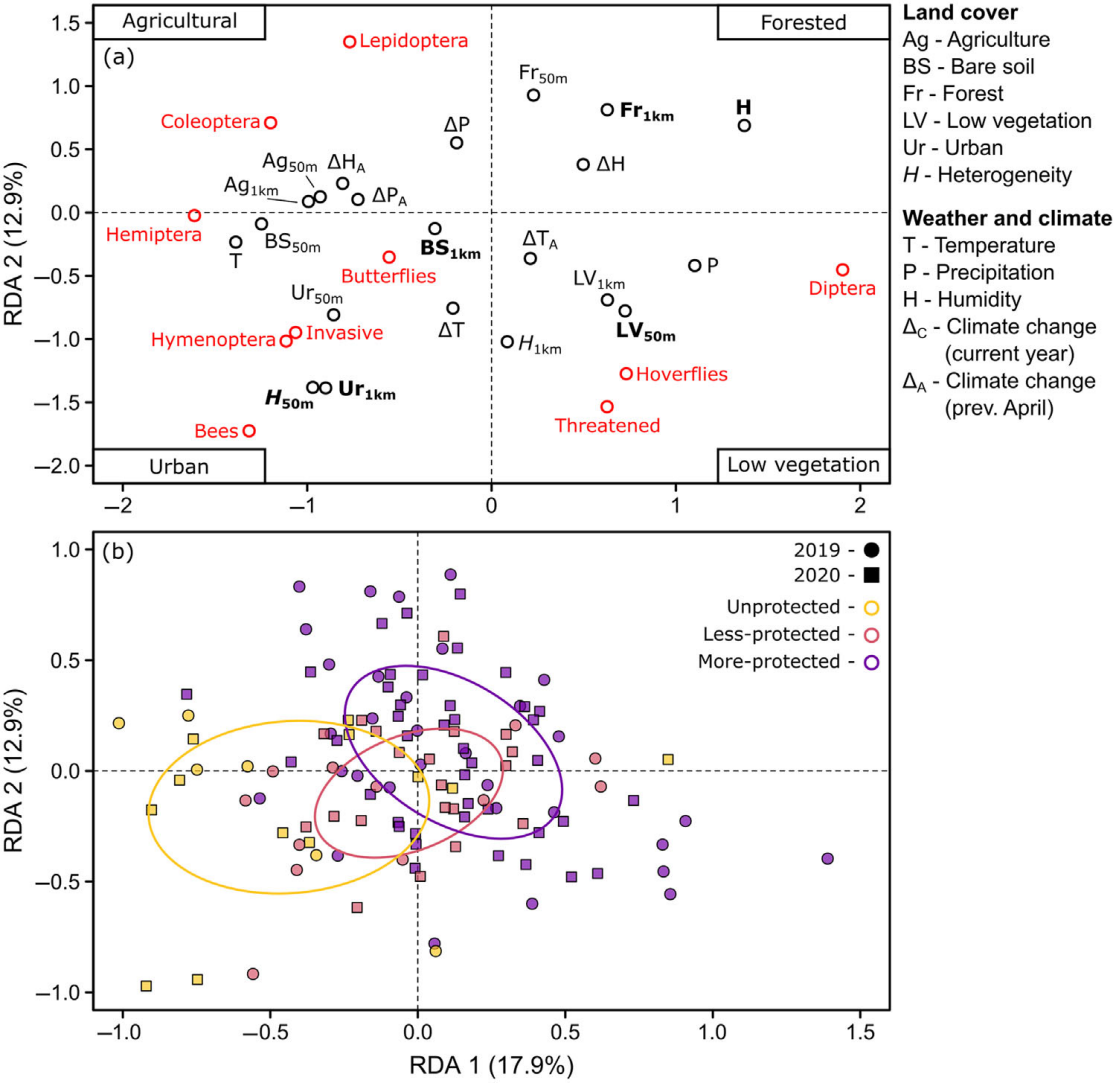
Redundancy analysis (RDA) of (a) the proportional richness of different insect groups (red points) in relation to land cover, weather, and climate (black points) (50 m, fine‐scale land cover; 1 km, broadscale land cover; bold type, predictors that consistently explained the most variation based on a stepwise model selection procedure [Appendix S14]) and (b) insect community composition of each site (points) (point colors, level of site protection; ellipses, central tendency for each protection category based on standard deviations). Pollinator groups (bees, butterflies, and hoverflies) are shown separately, and their corresponding orders (Hymenoptera, Lepidoptera, and Diptera) include these groups.

Based on the insect group (Figure 4), family‐level (Appendix S12), and trait composition RDAs (Appendix S12), communities at the low vegetation sites were characterized by certain Diptera families, such as hoverflies (lower right of Figure 4), and a mixture of multiple phytophagous guilds, such as miners (e.g., Agromyzidae) and stem feeders (e.g., Chloropidae), in addition to parasites (e.g., Tachinidae) (Appendix S12). Conversely, forested sites were characterized by a different set of primarily mycetophagous (i.e., fungus‐feeding) Diptera, such as fungus gnats (e.g., Mycetophilidae), and specific Lepidoptera families (right and upper sides of Figure 4), including certain moths (e.g., Adelidae or Geometridae). Composition was generally similar between urban and agricultural areas (both were located on the left side of Figure 4). However, there were some differences. For example, bees, butterflies, and invasive species accounted for greater proportions of communities in urban areas, where low vegetation was common (lower part of Figure 4) and there were more Coleoptera and Lepidoptera in agricultural areas with less low vegetation (upper left of Figure 4).

Sensitivity analyses indicated the above results were robust and not altered by excluding 11 traps under forest canopies (Appendix S2), where biomass and richness of flying insects might be underrepresented, or by the expansion of the monitoring network in 2020 to include 19 new sites (Figure 1; Appendix S13).

### Individual land‐cover, weather, and climate drivers (Q2)

Stepwise permutation tests of the land cover, weather, and climate predictors in the 4 RDAs indicated that 4 predictors always explained a significant portion of community variation, specifically low vegetation cover within a 50‐m radius, bare soil within 1 km, land‐cover heterogeneity within 50 m, and relative humidity, which were primarily represented along RDA axis 1 in all analyses (Figures 3 & 4; Appendices S12 & S14). Forest and urban cover within 1 km were also important in 3 out of the 4 RDAs (Appendix S14).

**FIGURE 5:**
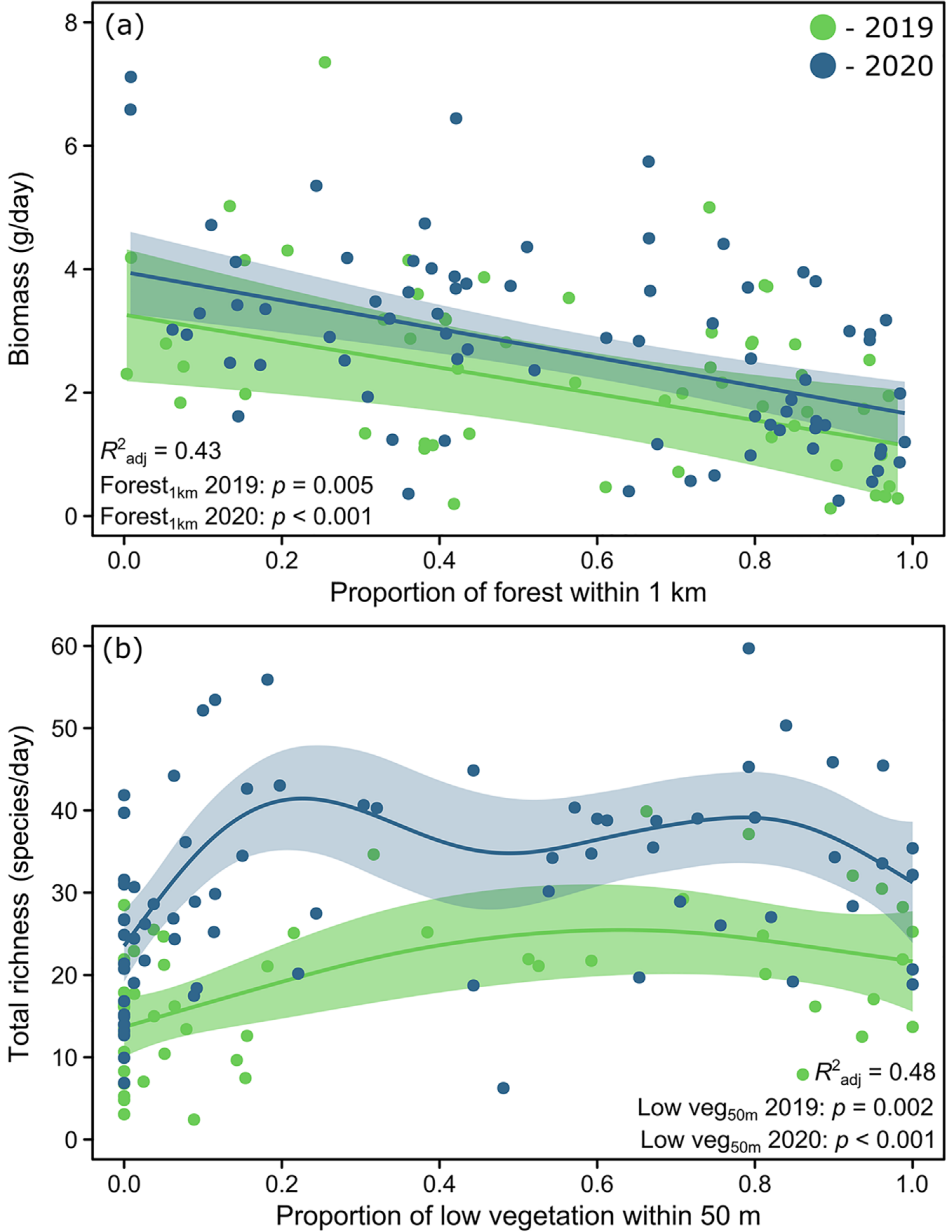
Relationship between (a) insect biomass and the proportion of forest cover within a 1‐km radius of traps and (b) total species richness and the proportion of low vegetation cover within a 50‐m radius of traps in 2019 and 2020. Modeled relationships are based on generalized additive model outcomes (solid lines, best fit; shading, 95% confidence intervals).

Based on GAMs, variability in biomass was best explained by forest cover within a 1‐km radius (*R* ^2^_adj_ = 0.23 out of a maximum of 0.41). All other predictors provided minor contributions (Appendix S15). Biomass tended to decrease by 50%, from 3.2 to 1.6 g/day, during 2019 as forest cover increased from 0% to 98% across sites (*n* = 56, edf = 1.0, *p* = 0.005). Similarly, it decreased by 56%, from 3.9 to 1.7 g/day, during 2020 (*n* = 75, edf = 1.0, *p* < 0.001) (Figure 5a).

Variability in total species richness was best explained by low vegetation cover within a 50‐m radius (*R* ^2^_adj_ = 0.39 out of a maximum of 0.48). Richness tended to increase by 58%, from 13.7 to 21.7 species/day, during 2019 as low vegetation cover increased from 0% to 99% across sites (*n* = 56, edf = 2.1, *p* = 0.002). There was a 33% increase, from 23.5 to 31.2 species/day, during 2020 (*n* = 73, edf = 4.6, *p* < 0.001) (Figure 5b). These relationships were not linear, and richness tended to plateau and even slightly decline once the proportion of low vegetation was above 50%.

### Protected areas (Q3)

We found differences among the 3 protected area categories in insect biomass and diversity (PERMANOVA, *n* = 123, *F*_2, 120_ = 11.1, *R* ^2^_adj_ = 0.16, *p* < 0.001) (Figure 3b), group composition (PERMANOVA, *n* = 123, *F*_2, 120_ = 10.7, *R* ^2^_adj_ = 0.15, *p* < 0.001) (Figure 4b), family‐level composition (PERMANOVA, *n* = 123, *F*_2, 120_ = 13.1, *R* ^2^_adj_ = 0.18, *p* < 0.001) (Appendix S12), and feeding trait composition (PERMANOVA, *n* = 123, *F*_2, 120_ = 7.1, *R* ^2^_adj_ = 0.11, *p* < 0.001) (Appendix S12). More‐protected sites were associated with humid forests, whereas unprotected sites were characterized by more low vegetation, urban, and agricultural cover and lower humidity. Less‐protected sites tended to be located between the more‐protected and unprotected sites in the RDAs, except for insect biomass and diversity where less‐protected and unprotected sites overlapped (Figure 3b). Given these associations, more‐protected sites were characterized by the same compositional patterns reported above for forested habitats, such as lower biomass and richness, higher turnover, and dominated by fungus‐feeding Diptera. Conversely, less‐protected and particularly unprotected sites were characterized by higher biomass, higher richness, lower turnover, and a mixture of insect groups and their associated feeding traits, including pollinators and threatened species.

## DISCUSSION

Periods (i.e., midsummer) and places with higher biomass exhibited higher richness and lower temporal turnover. The nature of the relationship between insect biomass and diversity is an open question (Hallmann et al., 2021; Hausmann et al., 2022; Uhler et al., 2021), particularly because studies reporting catastrophic insect declines often focus on overall biomass, but biomass loss may not equate to biodiversity loss. The matching patterns we found showed that, at least spatially, sites with lower biomass had a more depauperate insect community. The opposing relationships to temporal turnover also suggested lower biomass equated to higher temporal variation, which can increase community vulnerability to future disturbance (Hillebrand et al., 2018). Further research is needed to determine how these metrics relate to other community metrics of conservation interest, such as spatial turnover (i.e., spatial β diversity). For example, changes in insect biomass, richness, and composition can increase or decrease spatial β diversity depending on the affected species (Thorn et al., 2022), subsequently altering community stability and ecosystem functioning. Additionally, the relationships we found between biomass and other community metrics may be sensitive to the scale of study. For instance, changes in biomass can be less closely linked to richness or turnover if large‐bodied species comprise more of a given community, which could happen in less diverse areas (Hausmann et al., 2022) or when considering only a subset of taxa, such as bees (Vereecken et al., 2021). Consequently, biomass loss may indicate a loss of biodiversity and temporal stability when considered across many communities at broader spatial scales, but the same may not be true in more localized studies.

Our results on the spatial distribution of 31,846 flying insect species, specifically 10,803 validated plus 21,043 plausible species, primarily showed relationships with land cover, with increases in insect biomass (by 50% in 2019 and 56% in 2020) and species richness (by 58% and 33%) in relation to increases in low vegetation, such as grassland, meadows, gardens, and so forth. These relationships ran counter to our expectations that urban and agricultural areas, which tended to be dominated by low vegetation, would harbor fewer insects. The greater diversity of all insect groups with more low vegetation indicated that open areas may be biodiversity hotspots for a wide variety of flying insects. This inferred bolstering effect of low vegetation is supported by results of other research on individual insect groups, such as bees, whereby low vegetation benefits insect biomass and richness by diversifying nesting habitat and food resources (Mandelik et al., 2012; Wagner et al., 2019). Furthermore, the relationships we found applied broadly across a variety of insect taxa, suggesting the beneficial effects of low vegetation can apply to diverse groups of pollinating and nonpollinating flying insects, including beetles, flies, true bugs, wasps, butterflies, and moths. Enhancing the variety of forbs in low vegetation areas could therefore improve overall insect biomass and diversity in depauperate sites.

One possible explanation for the lower insect biomass and diversity in forests is that Malaise traps targeting flying insects can underrepresent other insect groups, including those under the forest canopy (Skvarla et al., 2021). However, this explanation is not completely satisfactory given we partly controlled for poor canopy sampling by placing most forest traps in gaps or along forest edges, following procedures used in other studies (Ganuza et al., 2022; Uhler et al., 2021). An alternative (or additional) explanation is that lower land‐cover heterogeneity in forests may reduce insect diversity. This interpretation is consistent with numerous studies on various insect and noninsect taxa showing that higher landscape heterogeneity can foster biodiversity, including in highly fragmented urban and agricultural landscapes, owing to higher habitat and resource diversity (Fahrig, 2017). Our feeding trait results support this explanation. Specifically, the dominance of forest communities by fewer feeding guilds than in other habitat types, including a prominence of fungus‐feeding flies, indicated a more homogeneous vegetation structure and resource supply. Conversely, the more even mixture of different plant feeding guilds in urban and agricultural areas indicated a more heterogeneous vegetation structure, likely facilitated by mixtures of natural vegetation (e.g., meadows, remnant forest patches) and anthropogenic vegetation (e.g., gardens, crops).

Our results showing homogeneous land cover in forests versus heterogeneous cover in urban and agricultural areas undoubtedly vary among studies and regions. For example, our surveyed sites captured a wide forest cover gradient, ranging from 0% to 100% cover at both finer and broader spatial scales, with many sites on both ends of the gradient. Consequently, sites with the highest forest cover were entirely dominated by trees, resulting in more homogenous land cover. In contrast, the urban and agricultural gradients were narrower and only a few sites were dominated (>50%) by these land‐cover types, meaning that most sites with higher urban or agricultural values exhibited a mixture of different land covers. Wider forest cover gradients versus narrower urban or agricultural gradients are not unusual in insect monitoring studies that rarely sample heavily urbanized or agricultural sites (Forister et al., 2023). More extensive sampling of such sites might have altered or even reversed our findings given that results of other studies show higher insect diversity in forests relative to heavily urbanized (Collado et al., 2019; Svenningsen et al., 2022) or agricultural areas (Ganuza et al., 2022; Uhler et al., 2021). Thus, seemingly opposite conclusions may be reached about the influence of certain land‐cover types on insects, depending on the extent of land‐cover gradients and associated habitat heterogeneity.

Although insect biomass and richness were lower in forests, these habitats supported unique communities of forest‐dwelling insects, such as fungus‐feeding flies, beetles, and moths. Forests are also often an underappreciated habitat for insects in nearby nonforested areas, such as pollinators or insects that depend on forests during parts of the year or particular life stages (Mola et al., 2021; Ulyshen et al., 2023). Consequently, our biomass and diversity results must not be misconstrued to mean that forests play a marginal role for insects. On the contrary, we found that forests contributed critically to regional diversity by supporting insects that do not occur elsewhere.

Although low to moderate urbanization did not reduce insect biomass or diversity, these sites exhibited more invasive species, suggesting potential negative impacts of urban centers on insect communities. Urban areas promote biological invasions by acting as hubs for the introduction and spread of non‐natives (Floerl et al., 2009) and offering unexploited niches for new species (Jesse et al., 2020). The horticultural trade is also a common vector for insect introductions (Smith et al., 2007), which could partly explain the association between urban areas and invasive insects given the greater presence of ornamental vegetation in lawns, gardens, and parks. These results highlight that, although low to moderate urbanization may increase habitat heterogeneity and thus benefit insect diversity, this effect can come with the detriment of promoting invasive insects. Such detriments could be mitigated by efforts to identify invasive species and control their spread (Francis & Chadwick, 2015) and by prioritizing native species in urban plantings (Tallamy et al., 2021).

We found no evidence for strong effects of temperature or precipitation on site‐level community differences, with only humidity exhibiting consistent relationships. Consistent humidity relationships likely reflect patterns in insect activity. Malaise traps record activity and abundance; thus, wetter conditions that reduce insect flight activity also reduce the number of captured specimens (Kaczmarek et al., 2022; Köthe, Schneider, et al., 2023), explaining why fewer insects were caught at higher humidity. The weaker effects we observed for temperature and precipitation may have occurred because spatial differences in these environmental factors can be less important for insects in temperate regions (Uhler et al., 2021). Responses may be stronger where insects are closer to their environmental limits, for example, in Mediterranean, tropical (Newbold et al., 2020), or higher elevation climate zones (Dalton et al., 2023). Our focus on within‐year spatial patterns may also emphasize the influence of land‐cover variability, which exhibits high variation at broad spatial scales, and deemphasize the influence of weather or climate, which may be more important at fine spatial scales (Colinet et al., 2015) or in temporal studies. For example, temperature effects on insects may be more detectable in long‐term temporal analyses across decades (Cardoso & Leather, 2019; Müller et al., 2023) or in seasonal analyses where the effects of short‐term extreme events can be more evident (Welti et al., 2022). The effects of temperature and precipitation may therefore become more evident as more spatial and temporal data are collected across regions.

Contrary to our expectations, overall biomass, richness, and richness of key functional groups (i.e., pollinators) and threatened insects tended to be as high or higher outside protected areas. Protected areas were primarily characterized by high forest cover and thus low insect biomass and diversity. This association with forests likely occurred because protected areas tend to be located in regions with little value for settlement and agriculture, such as forested, cooler, mountainous areas (Elsen et al., 2018; Joppa & Pfaff, 2009; Venter et al., 2018). These biases mean that protected areas can favor forest‐dwelling species (see similar patterns reported for birds in Cazalis et al. [2021]), and our results show this likely also applies to insects. Efforts to expand protected areas to meet the Kunming–Montreal Global Biodiversity Framework target of 30% protected land by 2030 (CBD, 2022) should therefore specifically consider insects in nonforested, relatively warm, low‐elevation areas. Protecting insects in such regions, which are often affected by urbanization and agriculture, may require establishing and expanding protected areas that allow for some human activity. This could include less‐protected areas, such as those in International Union for Conservation of Nature categories V and VI and the outer transition zones of biosphere reserves. However, the ecological benefits of these types of multi‐use protected areas are debated owing to inevitable trade‐offs with human needs (Dudley et al., 2010; Shafer, 2020). An additional option could be to prioritize alternative approaches to enhance insect diversity in unprotected areas, such as via urban green spaces (Anderson et al., 2023) and agroecological land management (Köthe, Bakanov, et al., 2023). Regardless of how best to achieve protection, our results clearly showed a high diversity of insects and important taxa outside forested protected areas, highlighting the need to consider insects when designating protected areas and to navigate trade‐offs between insect conservation and human needs.

The principal contribution of our study is the incorporation of a broad range of flying insect taxa when examining relationships among community metrics relative to environmental gradients. Across Germany, we found that low vegetation and land‐cover heterogeneity can simultaneously benefit total insect biomass, temporal stability, overall diversity, and the diversity of multiple taxonomic groups. Additionally, numerous species and key insects of conservation concern were unprotected because of existing biases in the designation of protected areas. These results highlight that conservation efforts aimed at expanding protected areas and improving insect habitat in urban and agricultural sites will not only benefit certain species (e.g., pollinators) but also likely benefit the broader insect community. Our work also showed why different studies can reveal different spatiotemporal patterns and drivers, such as the sampled gradient of urbanization and agriculture determining whether these stressors had positive or negative effects on insects. Likewise, the lack of weather and climate effects in our analyses suggested that spatially focused studies, such as ours, may increase the influence of land cover and decrease the importance of meteorological variables. In summary, our results highlight the value of a broader genetics‐based approach to identify key drivers of insect community change, inform future protection efforts, and reconcile different findings across studies. These broader perspectives are essential to understanding and mitigating insect biodiversity loss.

## Supporting information



Supporting information

Supporting information

Supporting information

Supporting information

Supporting information

Supporting information
