# Supplementary material for: Effects of land cover and protected areas on flying insect diversity
Source: Conserv Biol. 2024 Dec 4;39(4):e14425. doi: 10.1111/cobi.14425 (PMC12309638; doi:10.1111/cobi.14425)
Supplement: Supplementary file 2 — Supporting information [file COBI-39-e14425-s004.docx]

**Sinclair et al.** *Effects of land cover and protected areas on flying insect diversity*

**Appendix S2 –** Analyses without sites under forest canopies

Eleven Malaise traps were placed under forest canopies (listed in Table S2.1). These traps may miss insects flying in the canopy, so we repeated our analyses with these sites removed and found no substantial differences (Figures S2.1 and S2.2), so we provide the results with all sites included in the main text.

**Table S2.1.** List of 11 sites located underneath forest canopies. See Appendix S1 for the coordinates for all sites and their environmental characteristics.

| **Region name** | **Trap name** | **Coordinates**  (WGS 84 latitude, longitude) |
| --- | --- | --- |
| Bio-Hai | HEW-42 | 51.06991, 10.27354 |
| Bio-Sch-Alb | AEW-06 | 48.39412, 9.446531 |
| NP-BODD | Lang-2 | 54.44181, 12.49133 |
| NP-Eifel | Dedenborn | 50.56926, 6.359577 |
| NP-Eifel | K7 | 50.60815, 6.423185 |
| NP-Eifel | Lohrbachskopf | 50.59608, 6.464039 |
| NP-Eifel | Malsbenden | 50.57977, 6.467363 |
| NP-JAS | Fahrn-2 | 54.54618, 13.65944 |
| NP-Kel | 03 | 51.13155, 8.97643 |
| NP-SW | 02 | 48.68847, 8.241284 |
| NP-SW | 03 | 48.68475, 8.235532 |


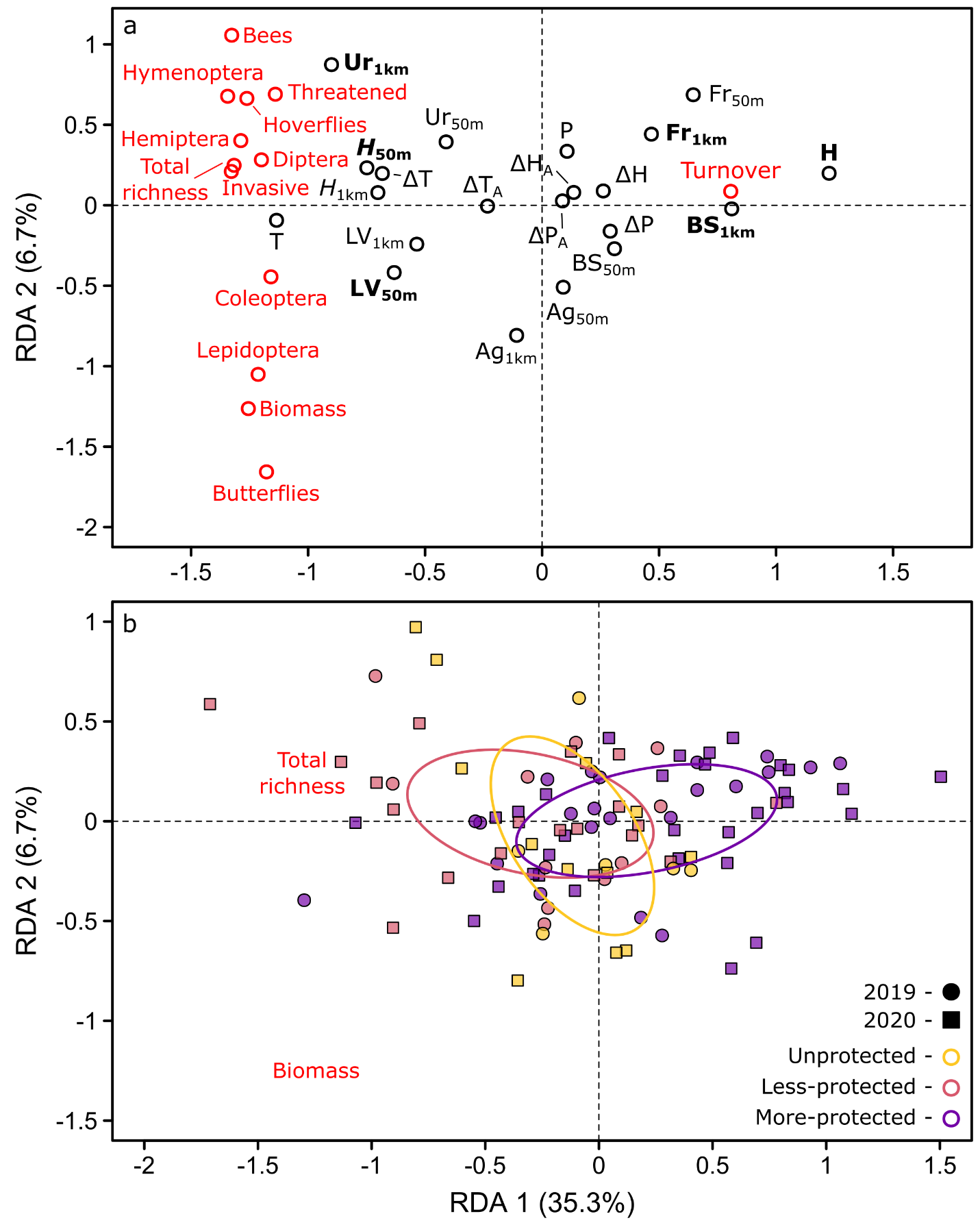


**Figure S2.1. Insect diversity for sites not under forest canopies.** Redundancy analysis (RDA) of (a) insect biomass, temporal turnover, total species richness, and the richness of different insect groups (red text) in relation to land cover, weather, and climate (black text) for only sites not under forest canopies (i.e., those in Table S2.1). Site locations on the RDA axes (shown in b; circles = 2019, squares = 2020) represent the similarity of their associated insect communities. Sites are colored by their protected area categories to illustrate community differences among these categories (purple = more protected, pink = less protected, yellow = unprotected), and colored ellipses indicate the central tendency for each category based on standard deviations. Predictor abbreviations are explained in the legend and Tables 1 and 2. Bold black text indicates predictors that consistently explained the most variation based on a stepwise model selection procedure (Appendix S14). Pollinator groups (bees, butterflies, and hoverflies) are shown separately, and their corresponding orders (Hymenoptera, Lepidoptera, and Diptera) include these groups.


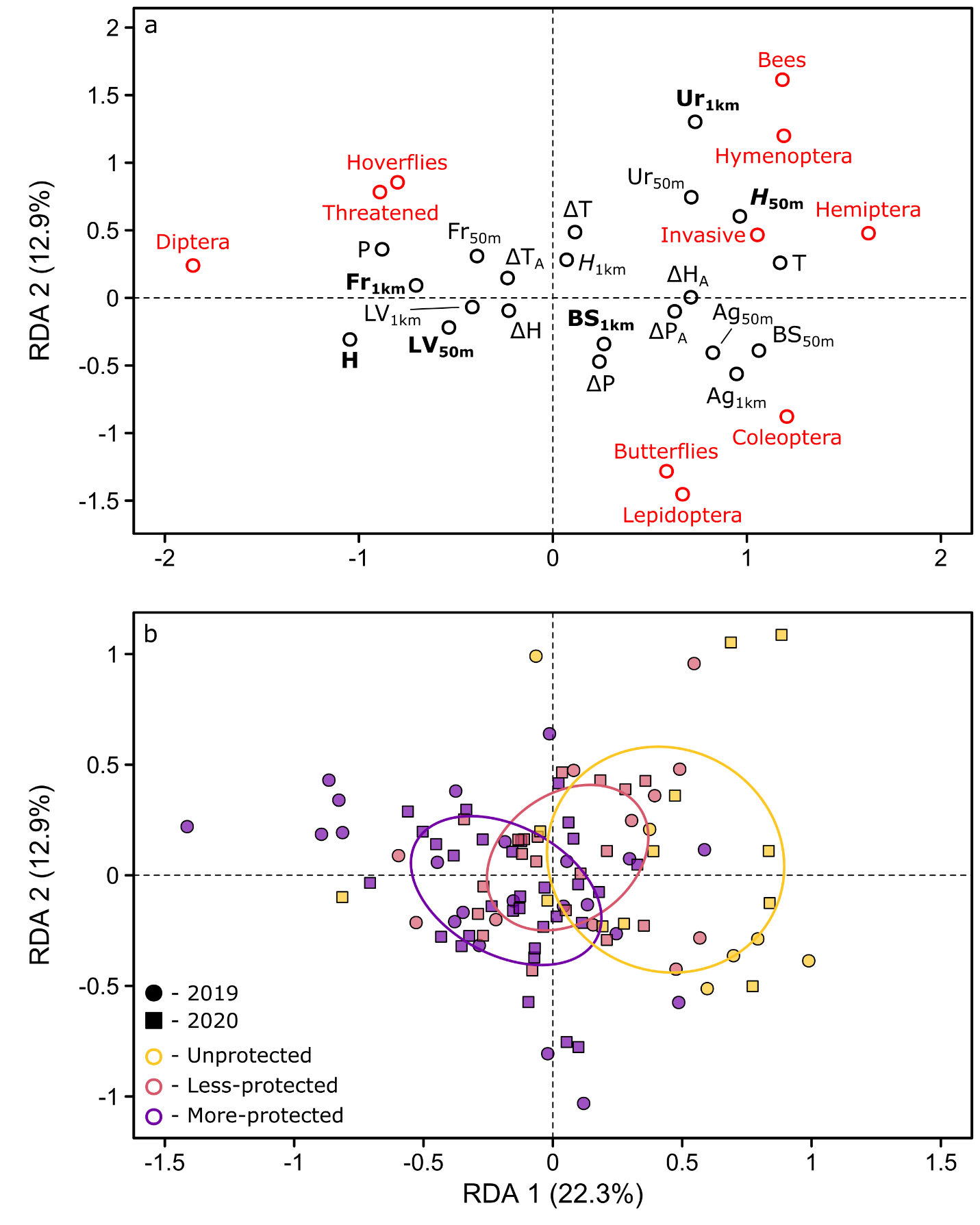


**Figure S2.2. Insect group composition from sites not under forest canopies.** Redundancy analysis (RDA) of (a) the proportional richness of different insect groups (red text) in relation to land cover, weather, and climate (black text) for only sites not under forest canopies (i.e., those in Table S2.1). Site locations on the RDA axes (shown in b; circles = 2019, squares = 2020) represent the similarity of their associated insect communities. Sites are colored by their protected area categories to illustrate community differences among these categories (purple = more protected, pink = less protected, yellow = unprotected), and colored ellipses indicate the central tendency for each category based on standard deviations. Predictor abbreviations are explained in the legend and Tables 1 and 2. Bold black text indicates predictors that consistently explained the most variation based on a stepwise model selection procedure (Appendix S14). Pollinator groups (bees, butterflies, and hoverflies) are shown separately, and their corresponding orders (Hymenoptera, Lepidoptera, and Diptera) include these groups.

**Appendix S3** – OTU richness

To estimate species richness from Operational Taxonomic Units (OTUs), we calculated the average number of OTUs per validated species for each insect family then divided the total number of OTUs per family by this number. For example, we identified 3,376 OTUs for the Ichneumonidae and an average of 1.2 OTUs per validated species. Based on this average, 3,376 OTUs equates to about 2,811 plausible species in this family. We repeated this process for each insect family. We then re-calculated total species richness as the sum of the plausible richness of all families. We also re-calculated the richness of the five major insect orders (Coleoptera, Diptera, Hemiptera, Hymenoptera, Lepidoptera) based on the plausible richness of the relevant families. Turnover was re-calculated as the change in richness among families, rather than species presence/absence, and so used the Sørensen index (with percent difference) rather than the Jaccard index. We did not include threatened or invasive species richness because these require species-level identifications.

The broad biodiversity and compositional results using plausible richness from OTUs were similar to those using only validated species. Biomass and richness for all taxonomic groups was higher in low vegetation areas (including urban and agricultural areas), and turnover was lower, with the opposite pattern in forested areas (Figure S3.1). The protected area patterns were also unchanged. These results match those reported in Figure 3. We did find some compositional differences, specifically a stronger association between forest cover and the proportional richness of Coleoptera, Hemiptera, and Lepidoptera, and a stronger association between low vegetation cover and butterflies (Figure S3.2). These altered associations likely occurred because OTU-based richness was higher for these taxonomic groups in these habitats, thus they comprised a greater proportion of total species richness. This suggests there may be some Coleoptera, Hemiptera, and Lepidoptera that are detected by OTUs but that cannot be resolved to validated species-level identifications. Note, however, that these differences are ultimately minor given that the principal land use, weather, and climate drivers are the same in all analyses and that the primary patterns are broadly similar.


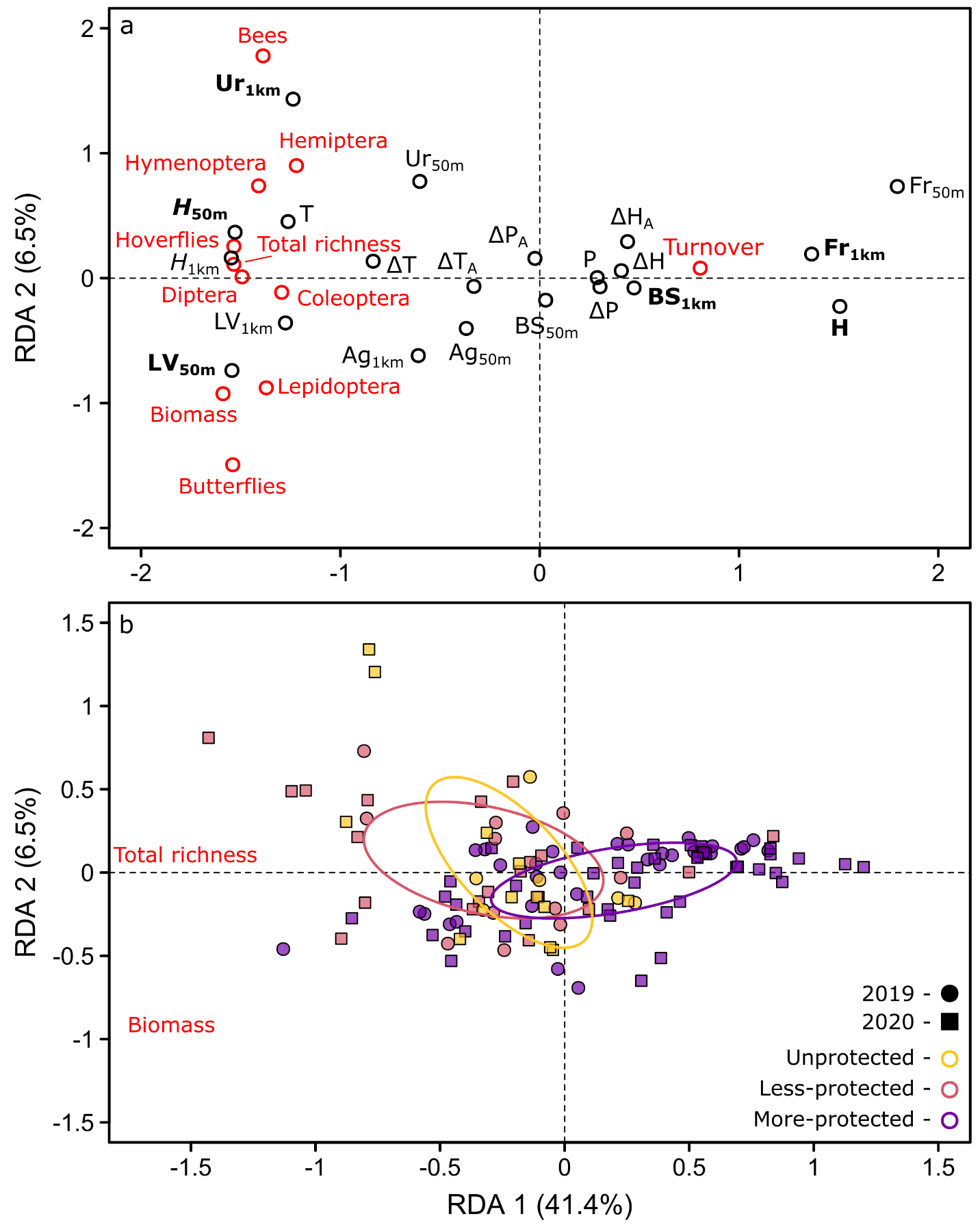


**Figure S3.1. Plausible insect diversity.** Redundancy analysis (RDA) of (a) insect biomass, temporal turnover, total species richness, and the richness of different insect groups (red text) in relation to land cover, weather, and climate (black text) for the plausible species dataset. Site locations on the RDA axes (shown in b; circles = 2019, squares = 2020) represent the similarity of their associated insect communities. Sites are colored by their protected area categories to illustrate community differences among these categories (purple = more protected, pink = less protected, yellow = unprotected), and colored ellipses indicate the central tendency for each category based on standard deviations. Predictor abbreviations are explained in Figures 3 & 4. Bold black text indicates predictors that consistently explained the most variation based on a stepwise model selection procedure (Appendix S14). Pollinator groups (bees, butterflies, and hoverflies) are shown separately, and their corresponding orders (Hymenoptera, Lepidoptera, and Diptera) include these groups. Threatened and invasive species were excluded because they require species-level identifications.


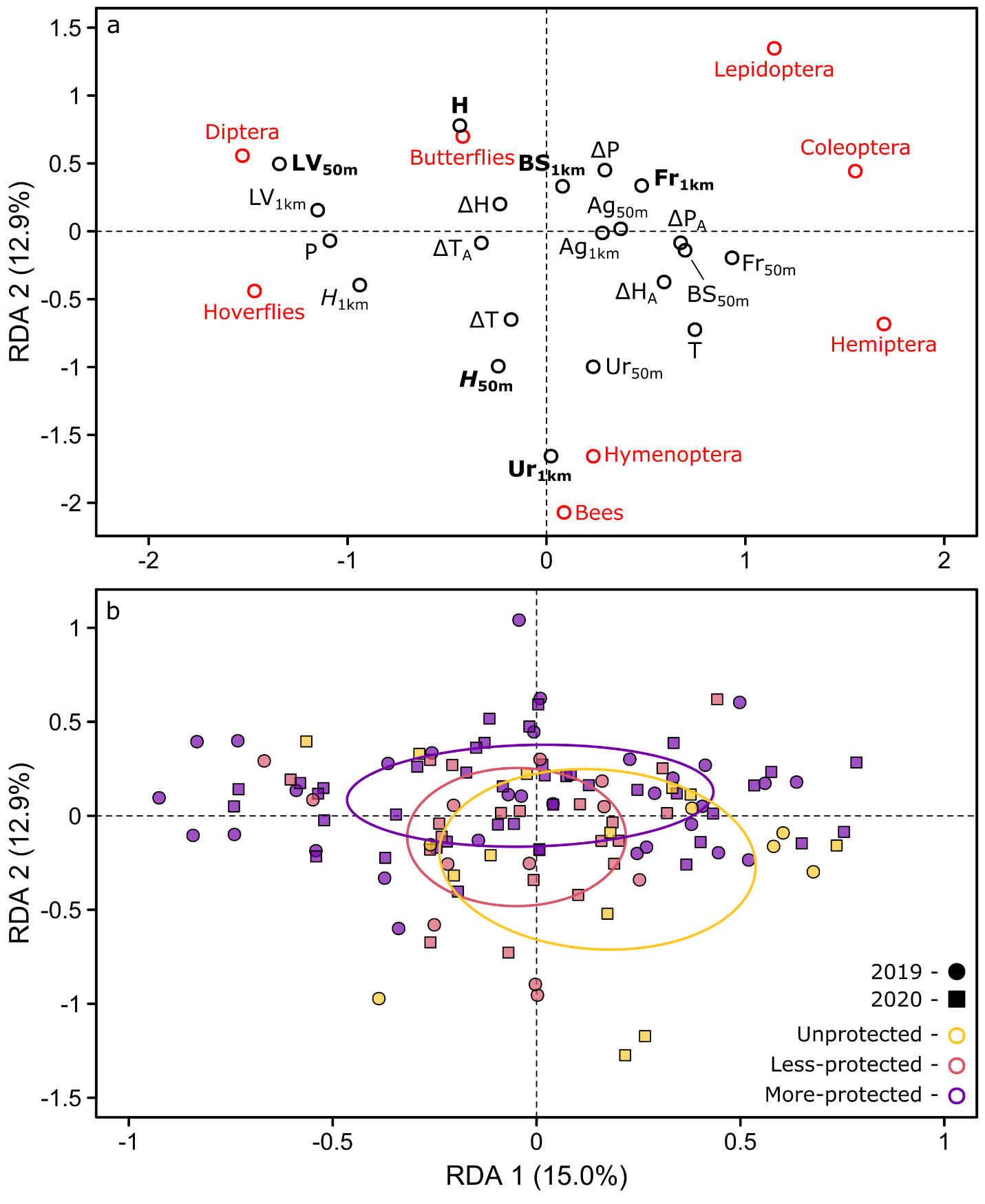


**Figure S3.2. Plausible group composition.** Redundancy analysis (RDA) of (a) the composition of plausible species from different insect groups (red text), expressed as proportional richness, in relation to land cover, weather, and climate (black text) for the plausible species dataset. Site locations on the RDA axes (shown in b; circles = 2019, squares = 2020) represent the similarity of their associated insect communities. Sites are colored by their protected area categories to illustrate community differences among these categories (purple = more protected, pink = less protected, yellow = unprotected), and colored ellipses indicate the central tendency for each category based on standard deviations. Predictor abbreviations are explained in Figures 3 & 4. Bold black text indicates predictors that consistently explained the most variation based on a stepwise model selection procedure (Appendix S14). Pollinator groups (bees, butterflies, and hoverflies) are shown separately, and their corresponding orders (Hymenoptera, Lepidoptera, and Diptera) include these groups. Threatened and invasive species were excluded because they require species-level identifications.

**Appendix S4 –** Insect families

**Table S4.1. Insect families.** List of insect families within groups comprised of multiple families, in addition to families considered to be ‘bees’, ‘butterflies’, and ‘hoverflies’.

| **Order** | **Common name** | **Families** |
| --- | --- | --- |
| Coleoptera | Fungus beetles | Corylophidae, Latridiidae |
|  | Weevils | Brentidae, Curculionidae |
| Diptera | Crane flies | Limoniidae, Tipulidae |
|  | Fruit flies | Drosophilidae, Tephritidae |
|  | Fungus gnats | Keroplatidae, Mycetophilidae, Sciaridae |
|  | Hoverflies | Syrphidae |
|  | Muscoids | Anthomyiidae, Fanniidae, Muscidae, Scathophagidae |
| Hemiptera | Planthoppers | Cixiidae, Delphacidae, Issidae |
|  | Psyllids | Psyllidae, Triozidae |
|  | Seed bugs | Heterogastridae, Lygaeidae, Rhyparochromidae |
| Hymenoptera | Bees | Andrenidae, Apidae, Colletidae, Halictidae, Megachilidae, Melittidae, Stenotritidae |
|  | Chalcid wasps | Eulophidae, Eurytomidae, Mymaridae, Torymidae |
|  | Crabronid wasps | Bembicidae, Pemphredonidae |
|  | Parasitic/parasitoid wasps | Aphelinidae, Braconidae, Diapriidae, Encyrtidae, Figitidae, Ichneumonidae, Platygastridae, Pteromalidae |
| Lepidoptera | Butterflies | Hesperiidae, Lycaenidae, Nymphalidae, Pieridae, Riodinidae |
|  | Gelechoid moths | Depressariidae, Elachistidae, Gelechiidae |
|  | Owlet moths | Erebidae, Noctuidae |
|  | Pyraloid moths | Crambidae, Pyralidae |

**Appendix S6** – Example weather gradients


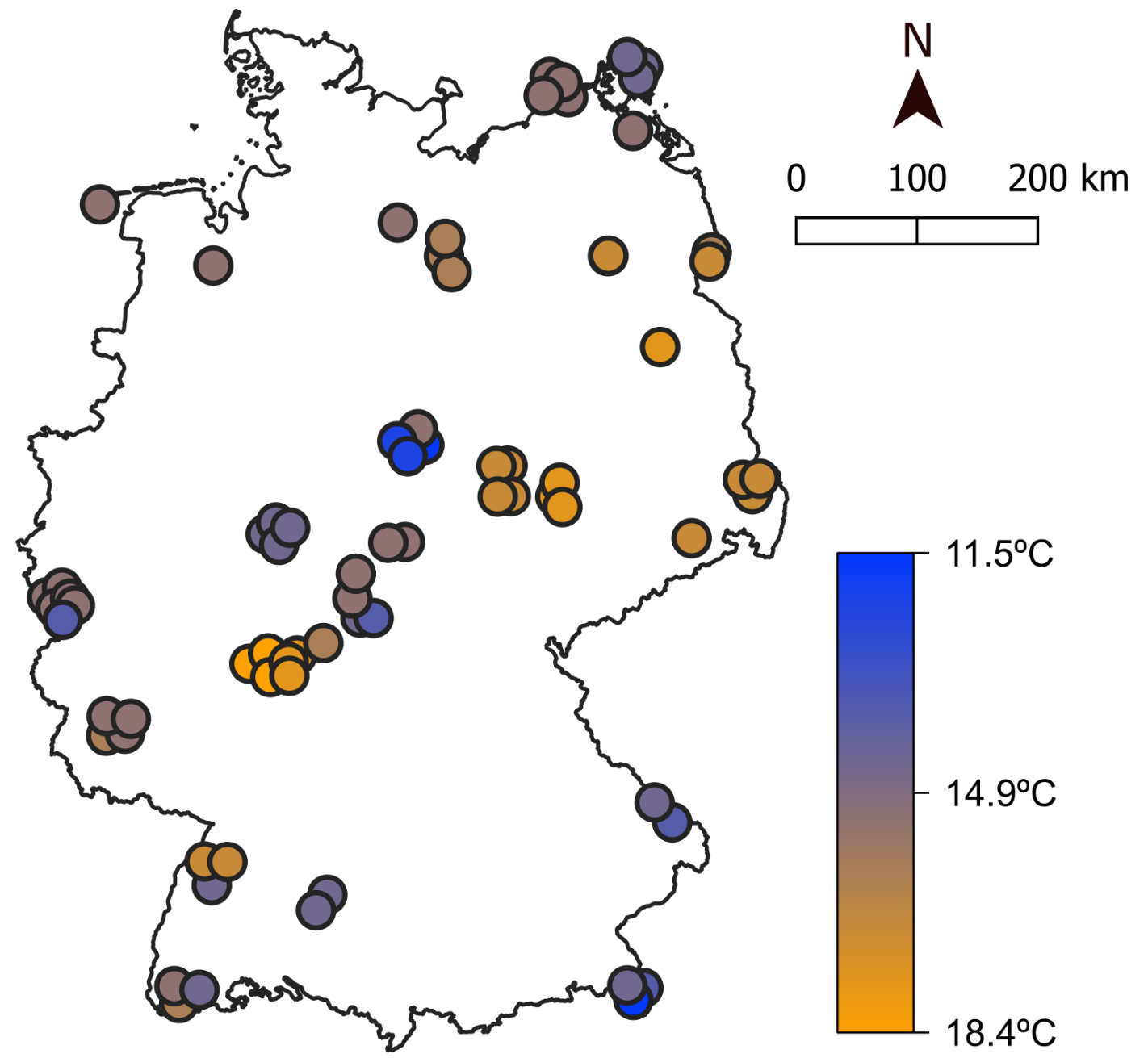


**Figure S6.1. Temperature in 2020.** Average of mean temperature (ºC) across all collection periods during late April to late September for each site in 2020. See Appendix S1 for site coordinates and all environmental characteristics.

**
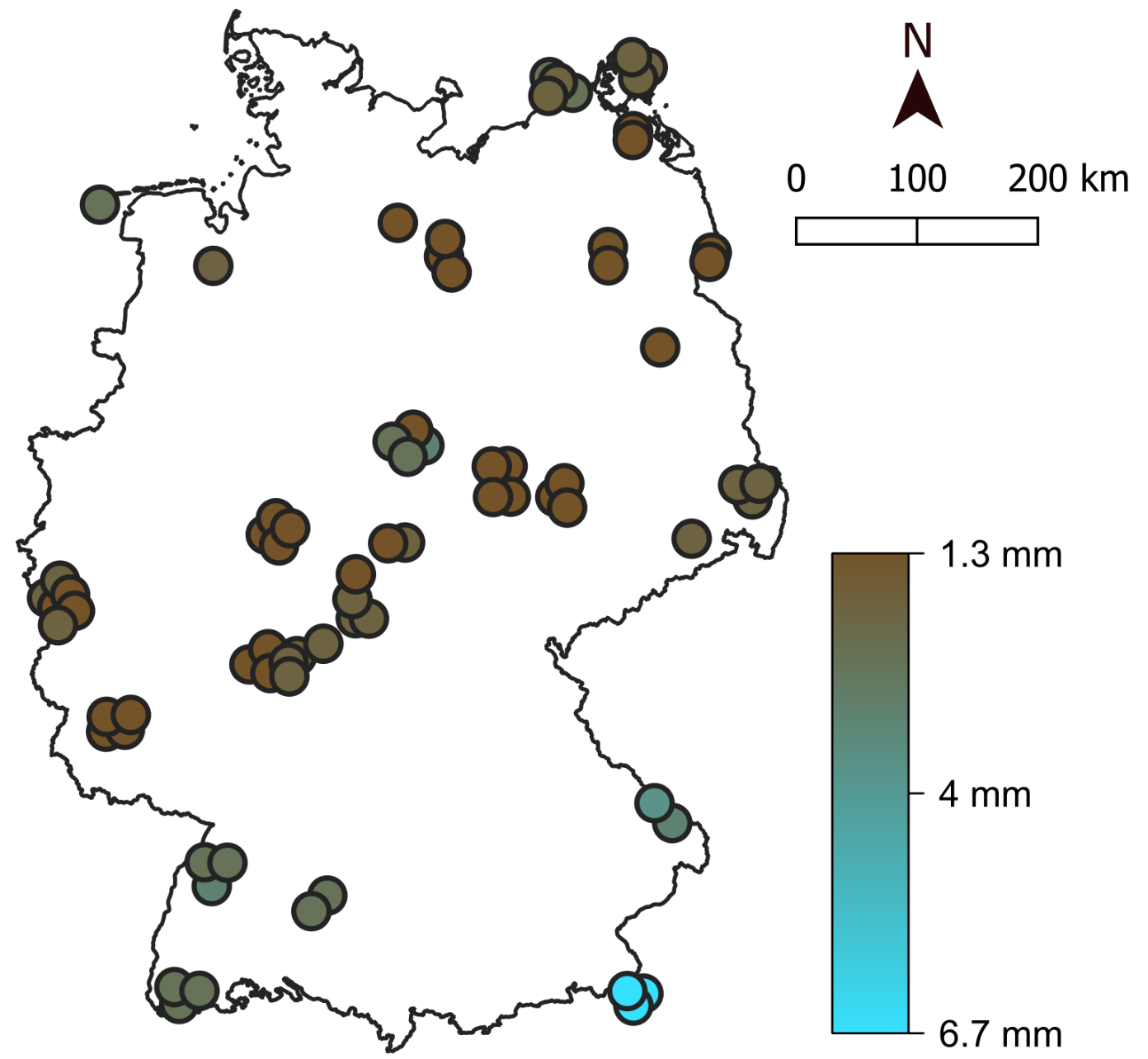
**

**Figure S6.2. Precipitation in 2020.** Average of total precipitation (mm) across all collection periods during late April to late September for each site in 2020. See Appendix S1 for site coordinates and all environmental characteristics.

**
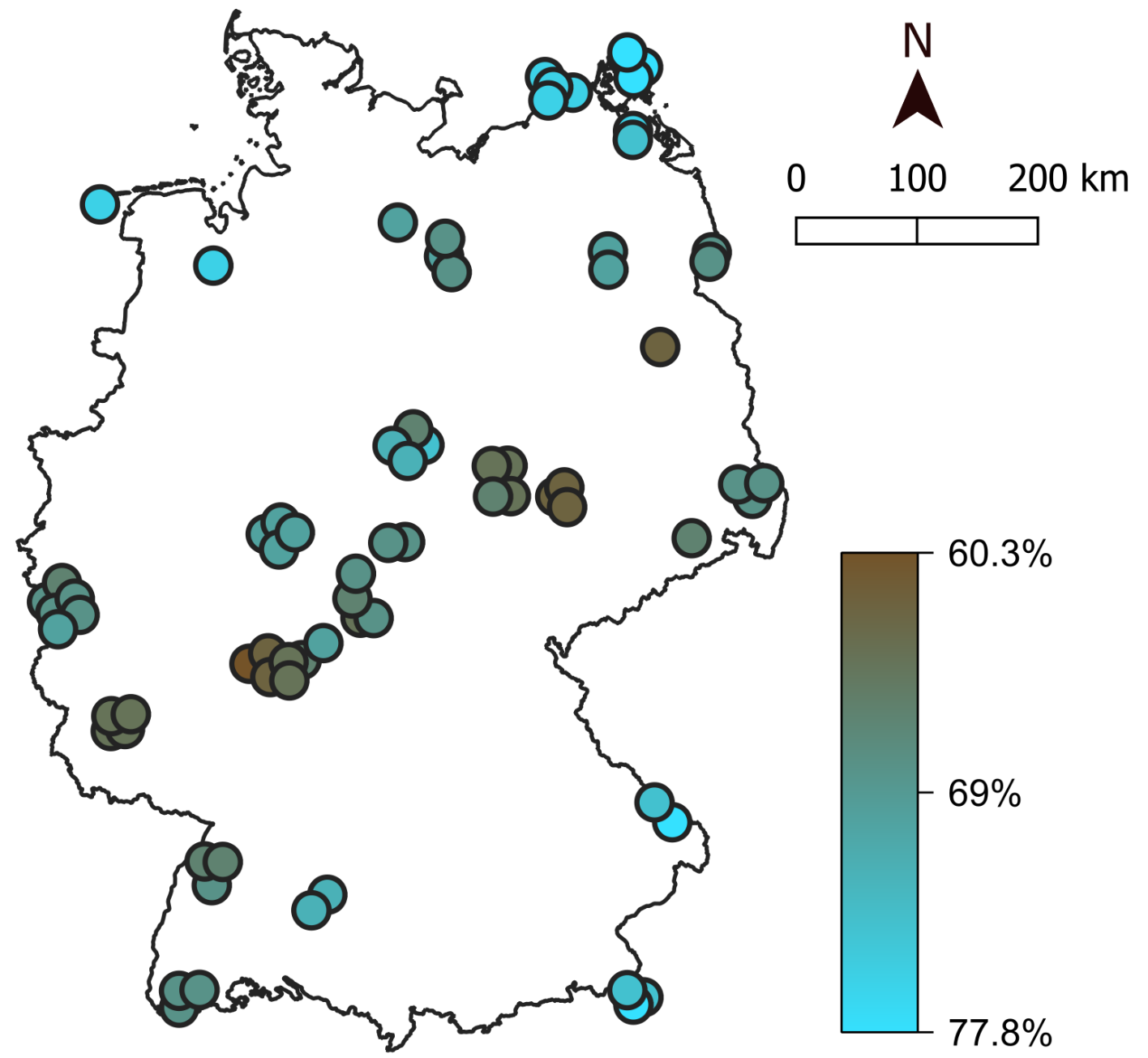
**

**Figure S6.3. Humidity in 2020.** Average of relative humidity (%) across all collection periods during late April to late September for each site in 2020. See Appendix S1 for site coordinates and all environmental characteristics.

**Appendix S10** – Seasonal patterns


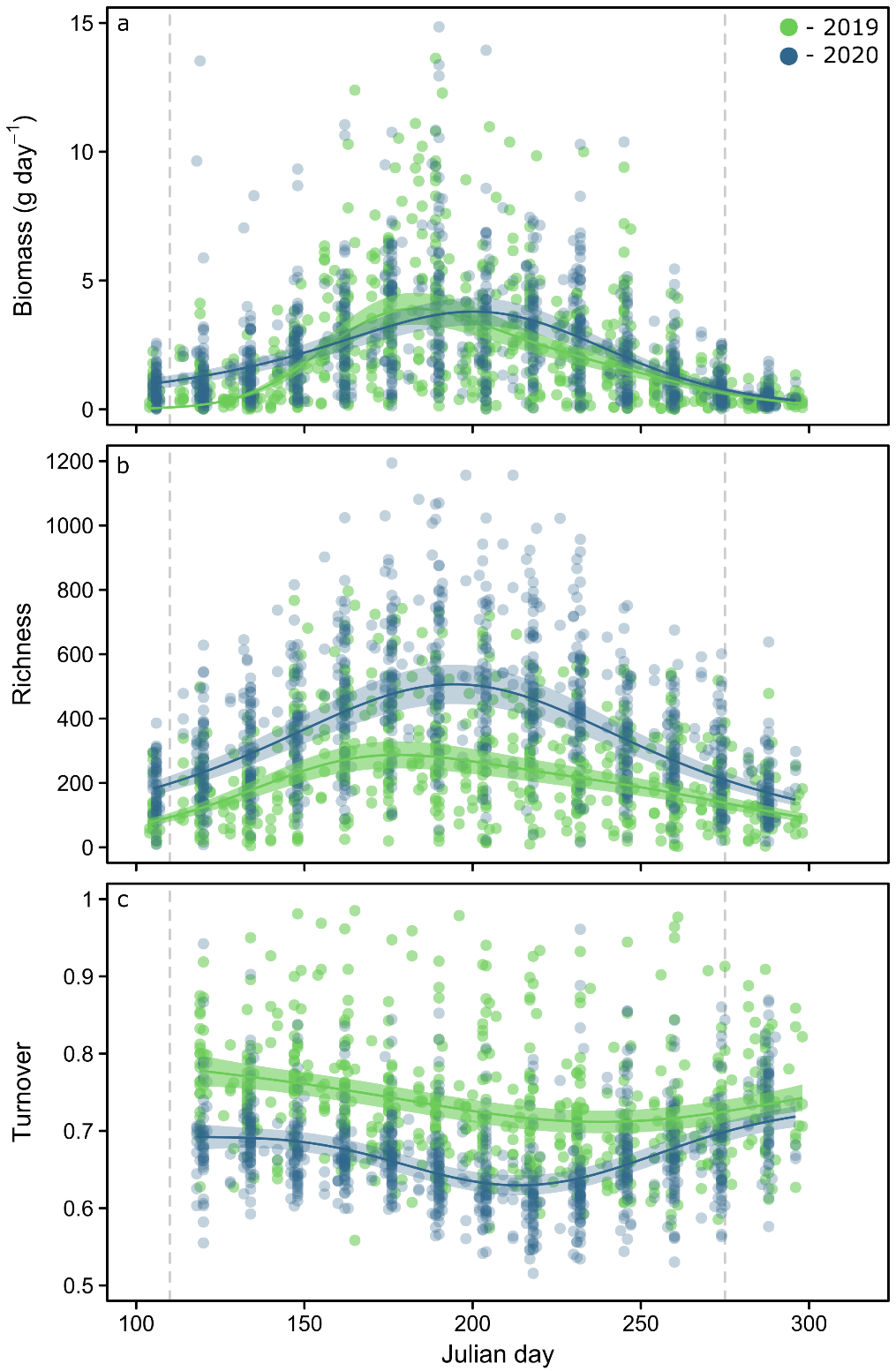


**Figure S10.1. Seasonal trends in biomass, richness, and temporal turnover.** Seasonal trends in (a) biomass (g day^-1^), (b) total species richness, and (c) temporal turnover (i.e., change in species composition between successive sampling periods) of flying insects caught in Malaise traps during the growing season in 2019 (green) and 2020 (blue). Best-fit lines (solid lines) and 95% confidence intervals (shaded areas) are based on GAMM estimates. Gray dashed lines indicate the period considered in all other analyses, specifically Julian days 110 (mid-April) to 273 (end of September).


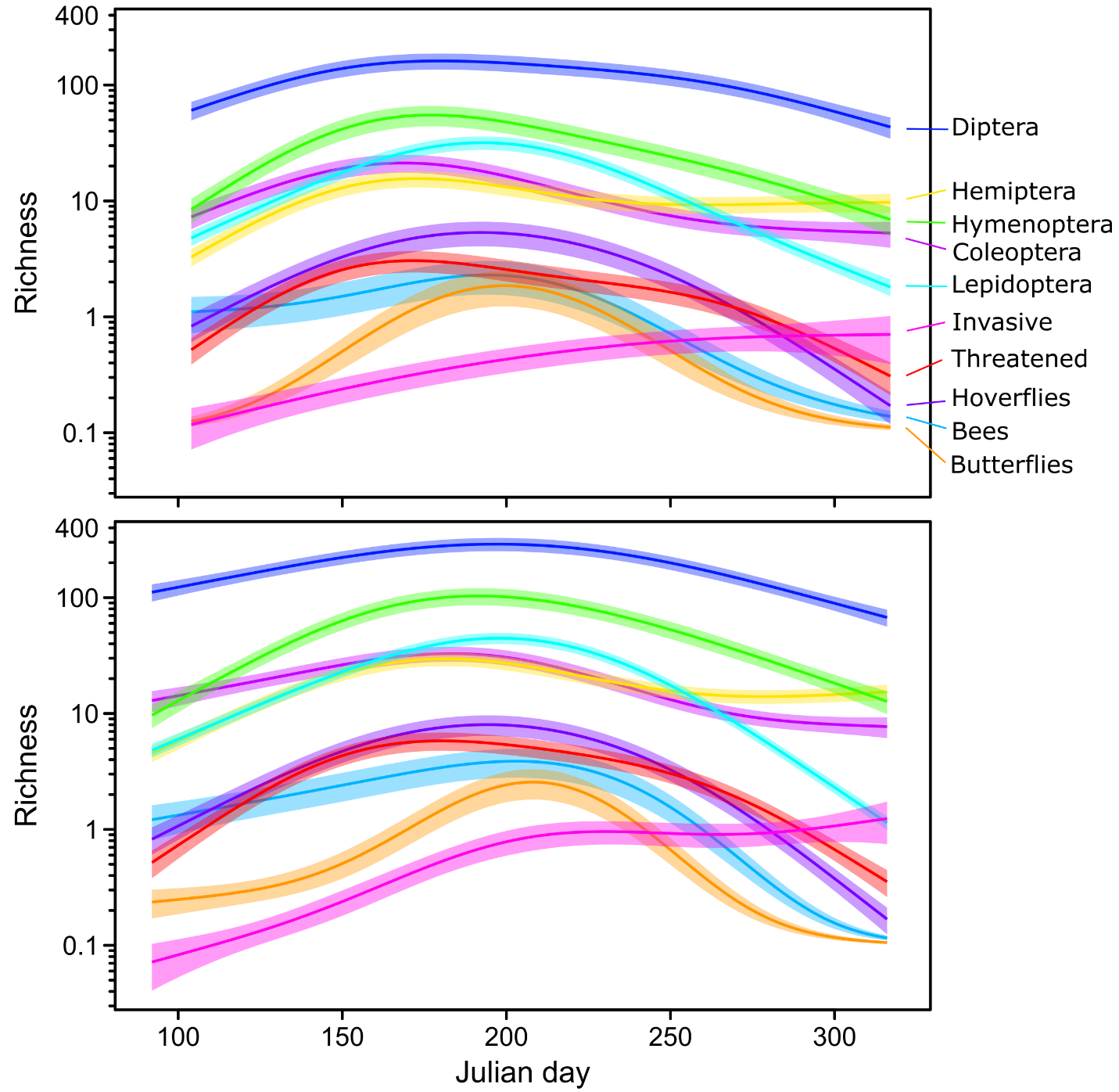


**Figure S10.2. Seasonal trends in taxonomic groups.** Seasonal patterns in species richness for the main insect groups during (a) 2019 and (b) 2020. Best-fit lines (solid lines) and 95% confidence intervals (shaded areas) are based on the respective generalized additive mixed model for each group. The invasive species are the only group exhibiting a different pattern compared to the others. This likely occurred because this group is comprised of fewer species and so its temporal patterns are more driven by the specific phenology of those species. The increase through the year is primarily driven by *Drosophila suzukii*, *Harmonia axyridis*, and somewhat *Leptoglossus occidentalis*. These species either tend to migrate towards the end of the year to find overwintering sites (e.g., *Drosophila suzukii*), and/or develop in larger numbers later in the year owing to higher prey availability (e.g., *Harmonia axyridis*), thus explaining why more are trapped later in the year.

**Appendix S11** – Variation partitioning


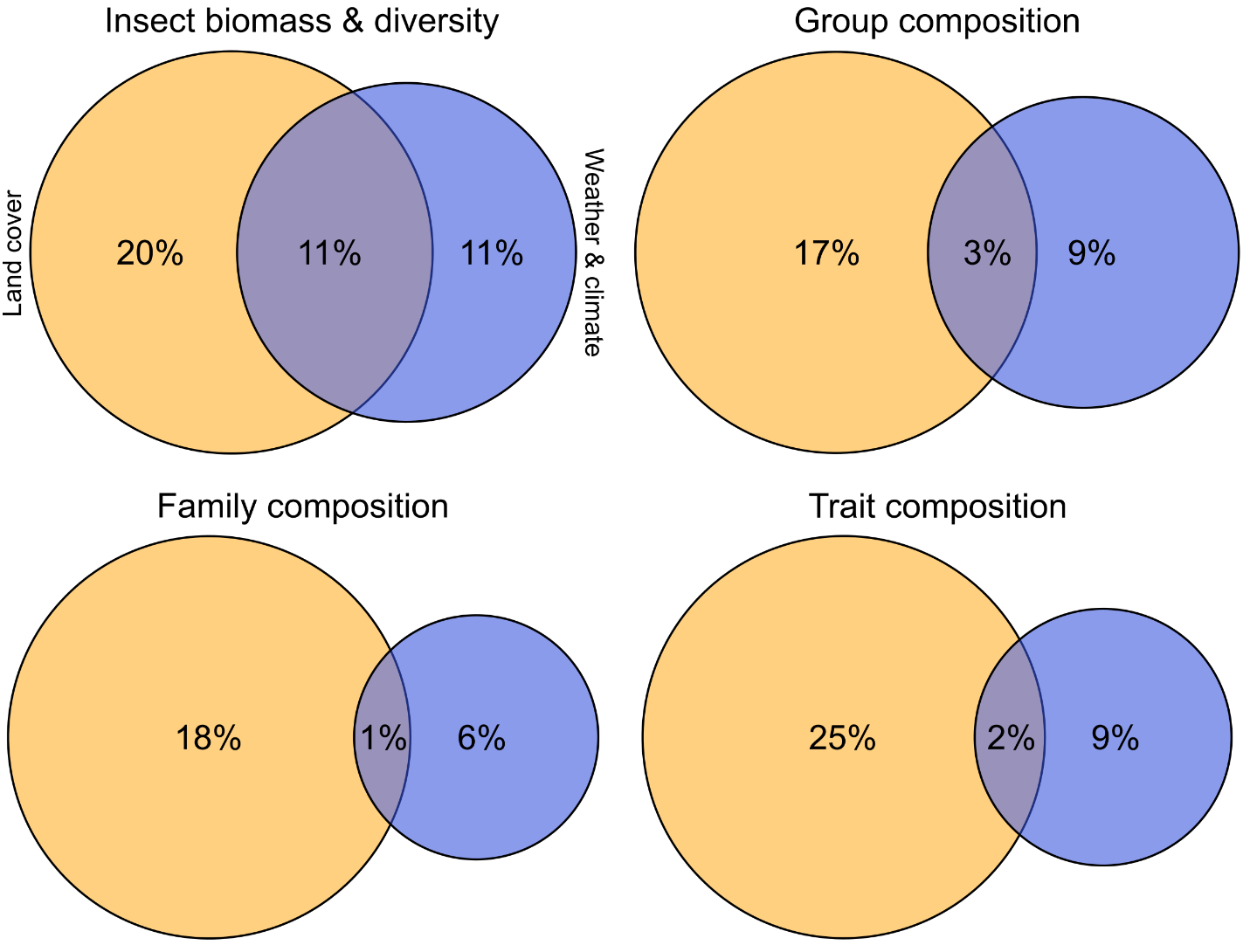


**Figure S11.1.** **Variation partitioning.** Percent of insect community variation explained by the individual effect of land cover (left circle; orange), the individual effect of weather and climate (right circle; blue), and their covariation (overlapping area; purple).

**Appendix S12 –** Insect family-level and feeding trait composition


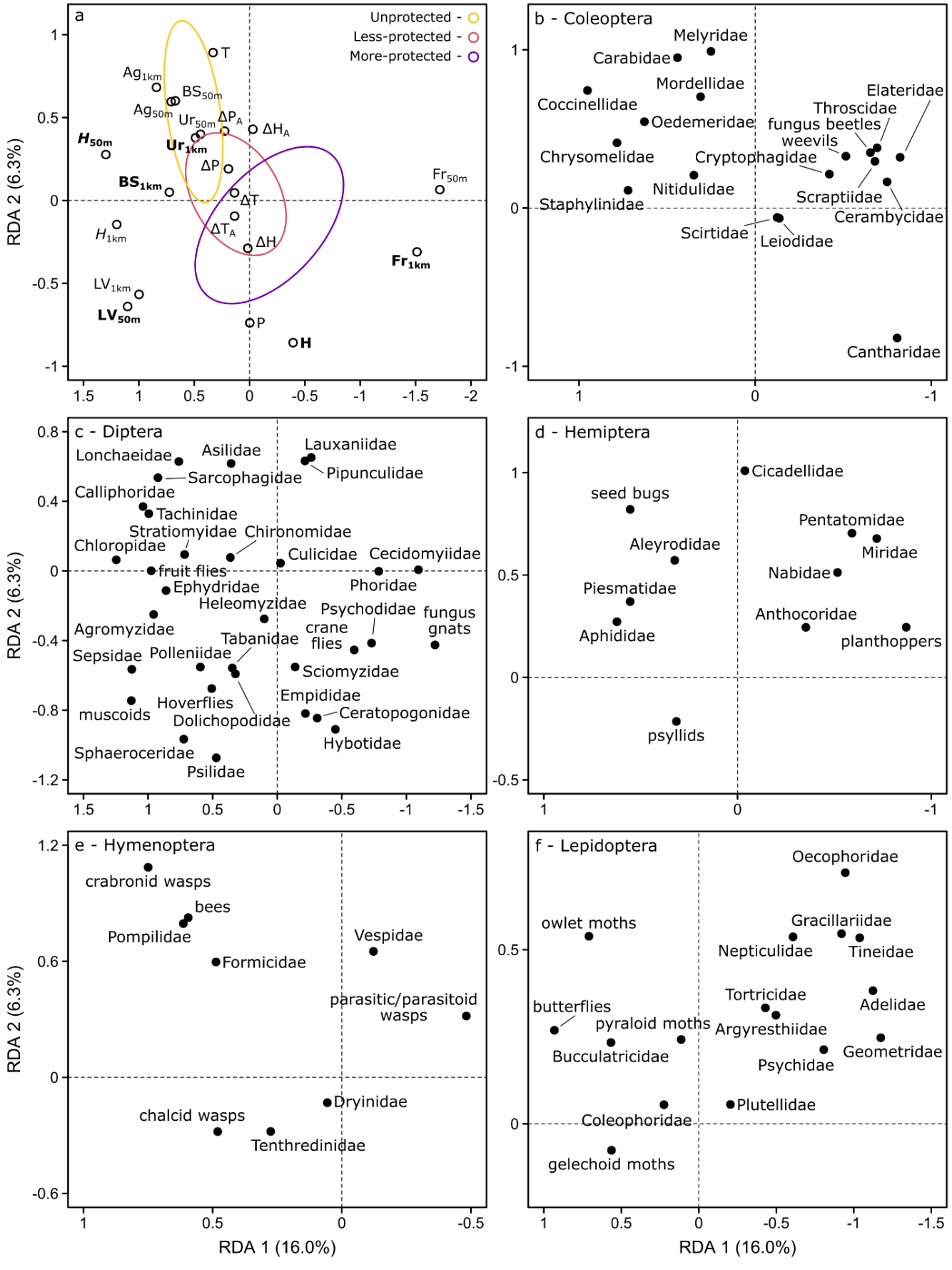


**Figure S12.1. Insect family-level composition.** Redundancy analysis (RDA) of (a) insect family-level composition in relation to land cover, weather, and climate. Site locations are not shown for simplicity, but colored ellipses in panel a indicate the central tendency for the three protected area categories based on standard deviations. Predictor abbreviations are explained in Figures 3 & 4. Bold black text indicates predictors that consistently explained the most variation based on a stepwise model selection procedure (Appendix S14).


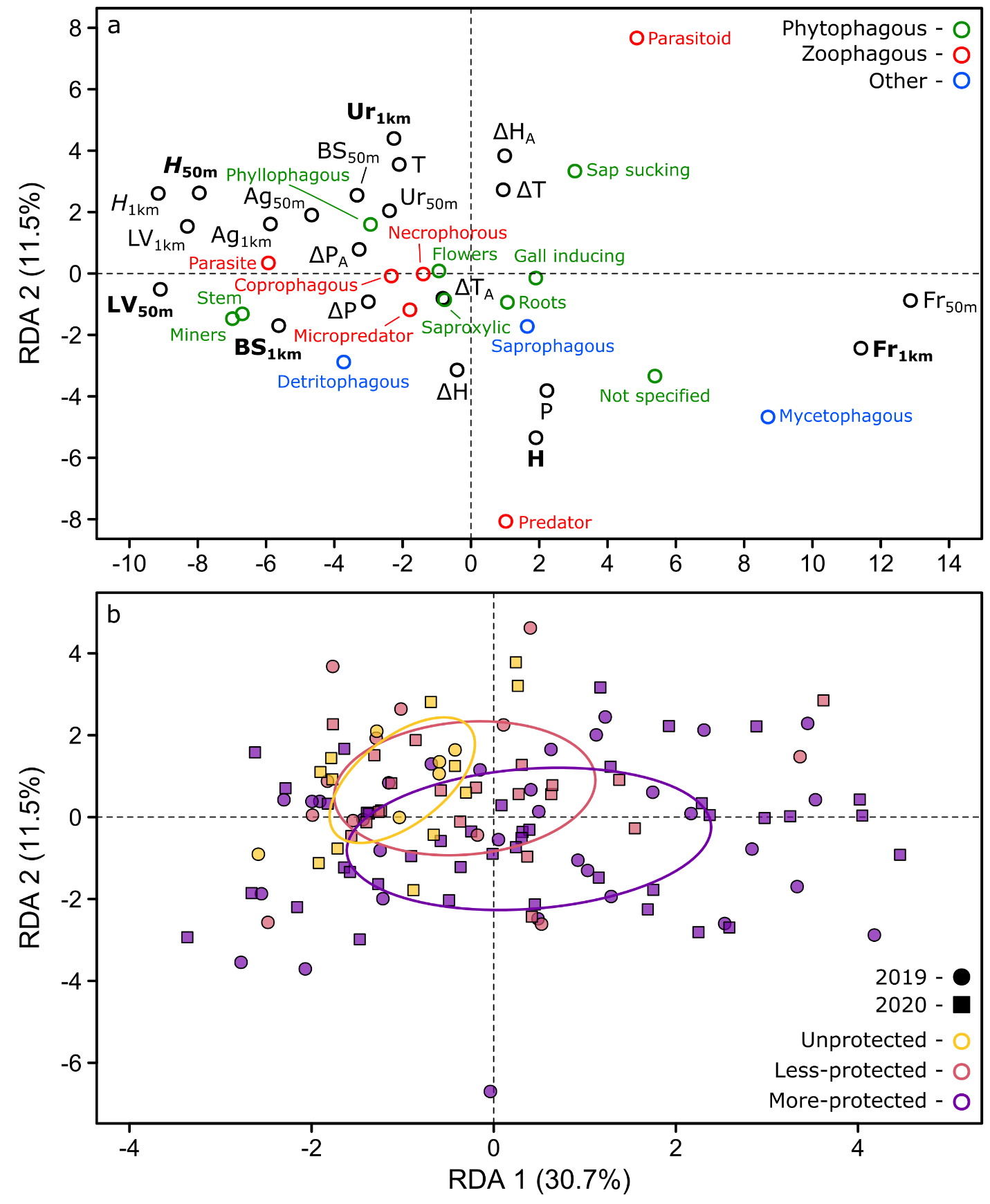


**Figure S12.2. Insect trait composition.** Redundancy analysis (RDA) of (a) insect feeding trait composition (colored text) in relation to land cover, weather, and climate (black text). Site locations on the RDA axes (shown in b; circles = 2019, squares = 2020) represent the trait similarity of their associated insect communities. Sites are colored by their protected area categories to illustrate community differences among these categories (purple = more protected, pink = less protected, yellow = unprotected), and colored ellipses indicate the central tendency for each category based on standard deviations. Predictor abbreviations are explained in Figures 3 & 4. Bold black text indicates predictors that consistently explained the most variation based on a stepwise model selection procedure (Appendix S14). Pollinator groups (bees, butterflies, and hoverflies) are shown separately, and their corresponding orders (Hymenoptera, Lepidoptera, and Diptera) include these groups.

**Appendix S13** – Only sites sampled in both 2019 and 2020


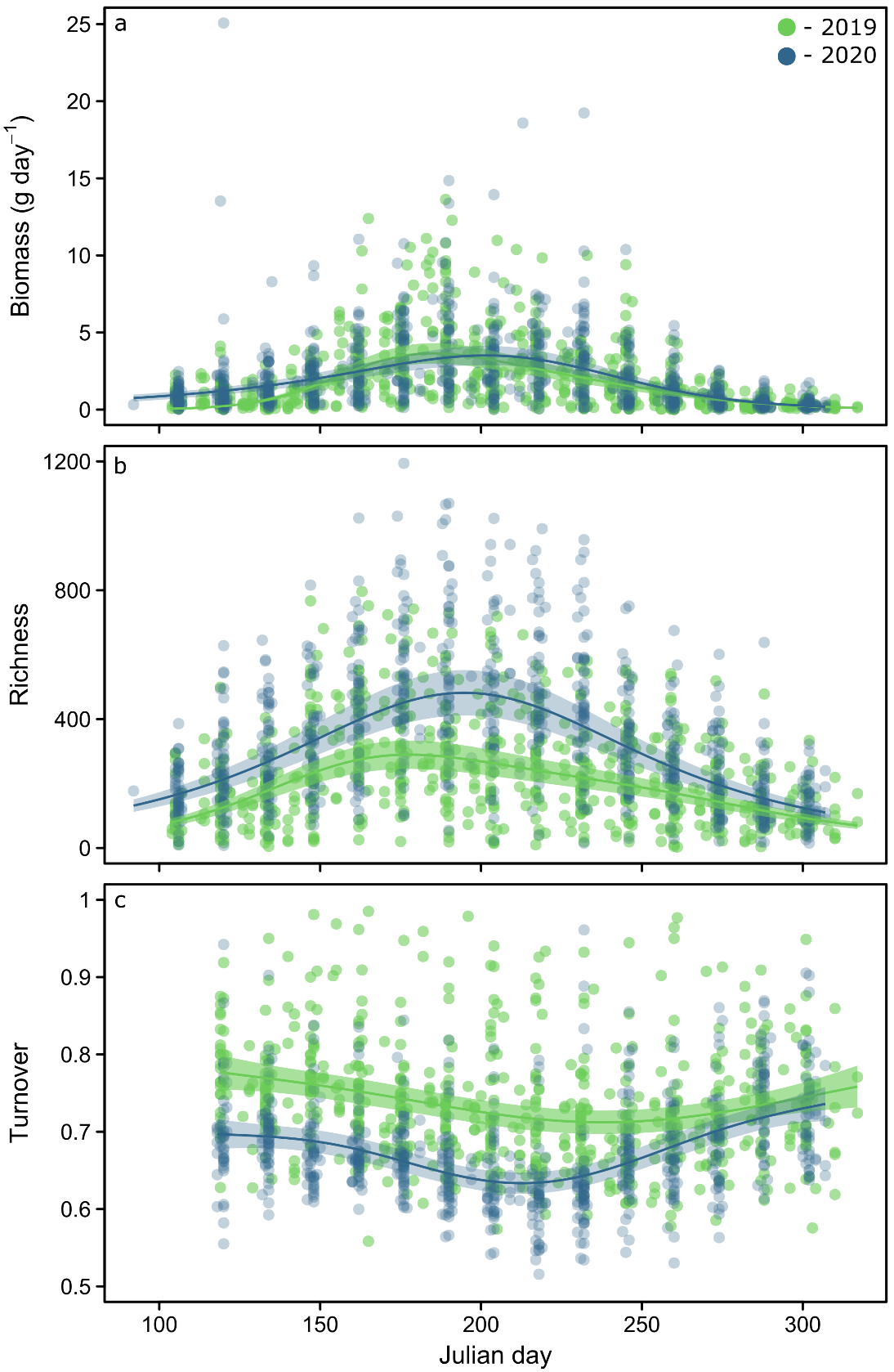


**Figure S13.1. Seasonal trends only for sites sampled in both 2019 and 2020.** Seasonal patterns in (a) biomass (g day^-1^), (b) total species richness, and (c) temporal turnover, but only for traps sampled during both 2019 (orange) and 2020 (purple). Best-fit lines (solid lines) and 95% confidence intervals (shaded areas) are based on estimates from their respective generalized additive mixed models.

**
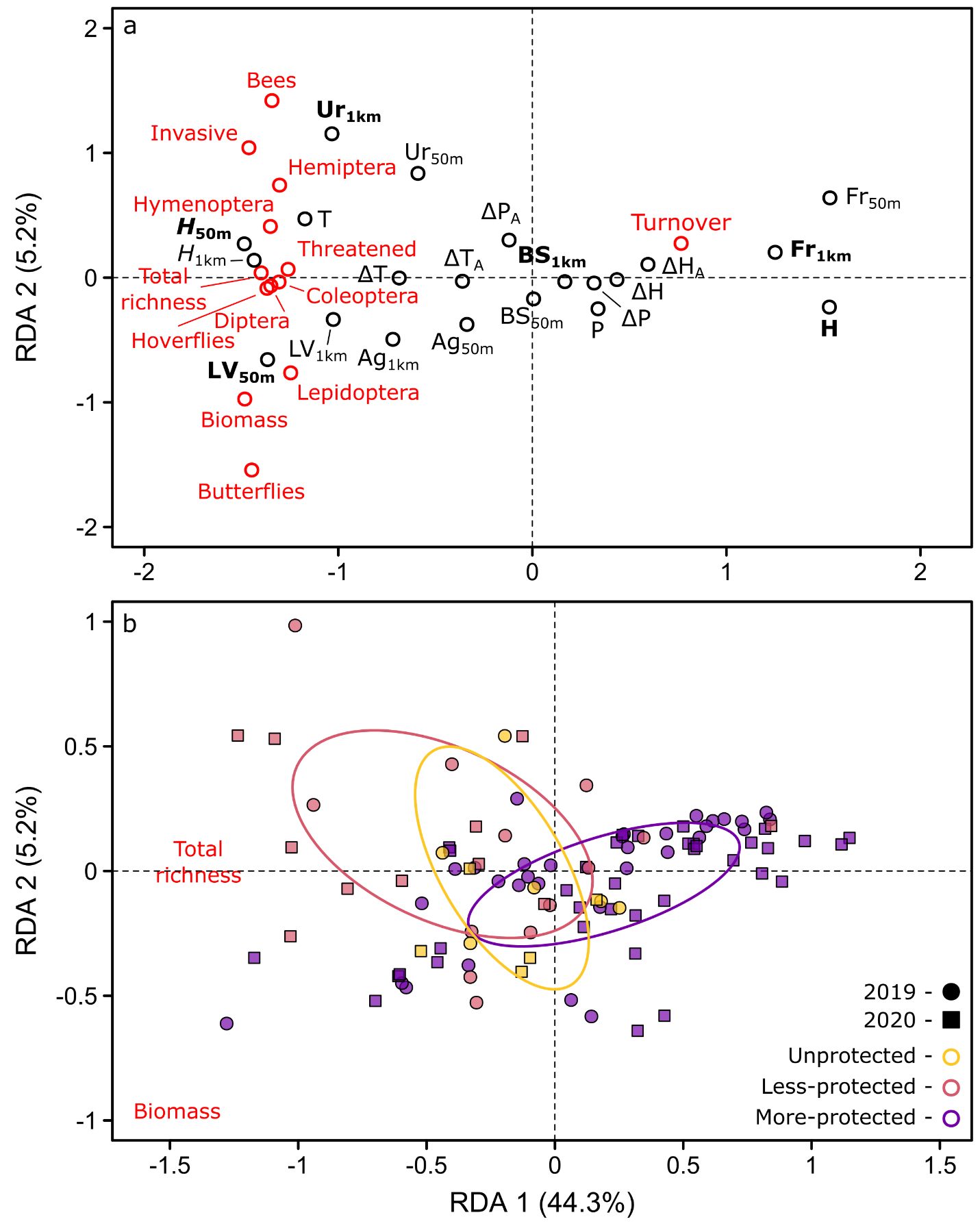
**

**Figure S13.2. Insect diversity only for sites sampled in both 2019 and 2020.** Redundancy analysis (RDA) of (a) insect biomass, temporal turnover, total species richness, and the richness of different insect groups (red text) in relation to land cover, weather, and climate (black text) for only sites sampled in both 2019 and 2020. Site locations on the RDA axes (shown in b; circles = 2019, squares = 2020) represent the similarity of their associated insect communities. Sites are colored by their protected area categories to illustrate community differences among these categories (purple = more protected, pink = less protected, yellow = unprotected), and colored ellipses indicate the central tendency for each category based on standard deviations. Predictor abbreviations are explained in the legend and Figures 3& 4. Bold black text indicates predictors that consistently explained the most variation based on a stepwise model selection procedure (Appendix S14). Pollinator groups (bees, butterflies, and hoverflies) are shown separately, and their corresponding orders (Hymenoptera, Lepidoptera, and Diptera) include these groups.


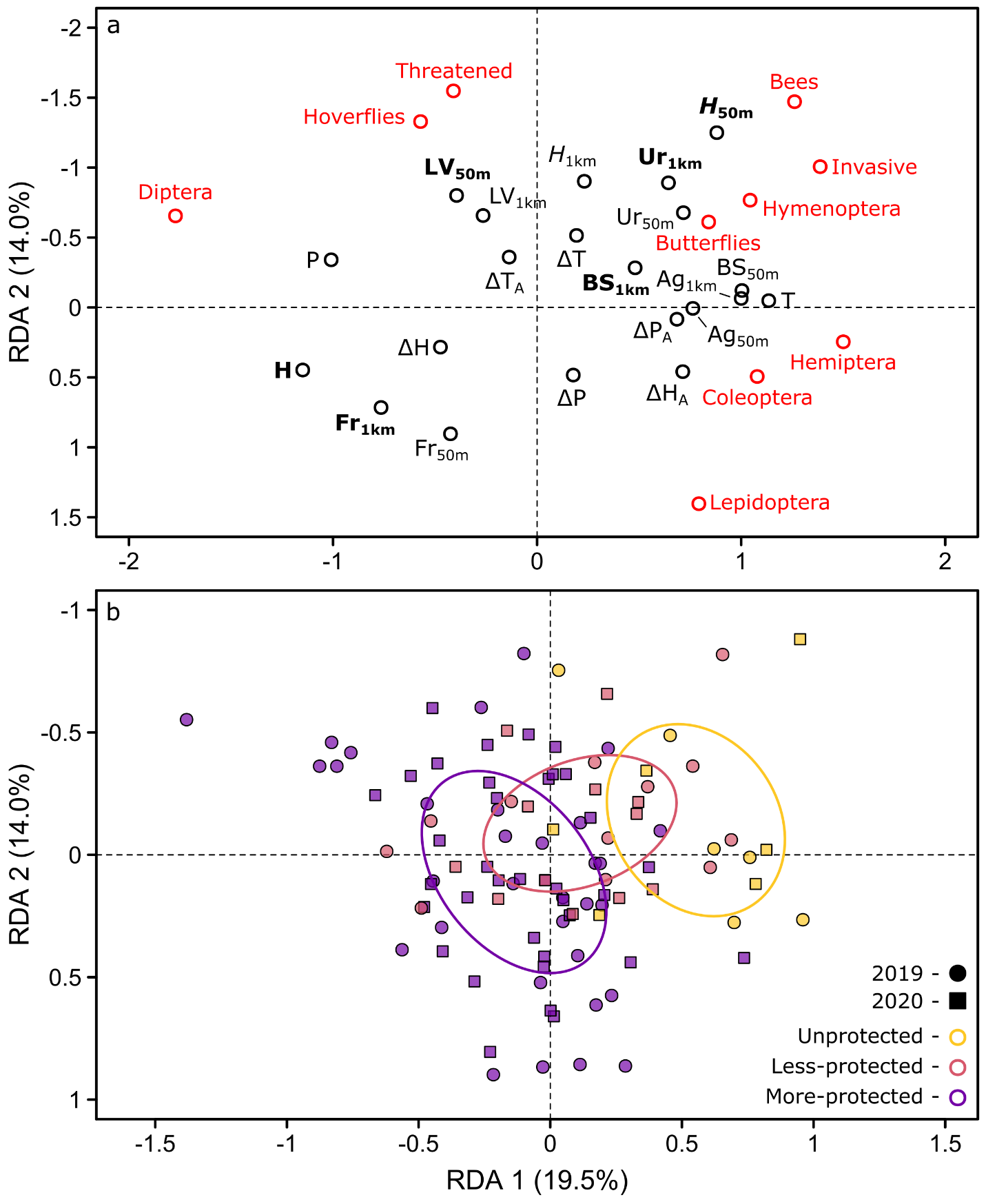


**Figure S13.3. Insect group composition only for sites sampled in both 2019 and 2020.** Redundancy analysis (RDA) of (a) the proportional richness of different insect groups (red text) in relation to land cover, weather, and climate (black text) for only sites sampled in both 2019 and 2020. Site locations on the RDA axes (shown in b; circles = 2019, squares = 2020) represent the similarity of their associated insect communities. Sites are colored by their protected area categories to illustrate community differences among these categories (purple = more protected, pink = less protected, yellow = unprotected), and colored ellipses indicate the central tendency for each category based on standard deviations. Predictor abbreviations are explained in the legend and Figures 3 & 4. Bold black text indicates predictors that consistently explained the most variation based on a stepwise model selection procedure (Appendix S14). Pollinator groups (bees, butterflies, and hoverflies) are shown separately, and their corresponding orders (Hymenoptera, Lepidoptera, and Diptera) include these groups.

**Appendix S14 –** Important individual predictors

**Table S14.1.** Six predictors consistently explained the most variation across four RDAs of: (i) insect biomass and diversity; (ii) group composition; (iii) family-level composition; and (iv) feeding trait composition.

| **Predictor** | **Insect biomass & diversity** | **Group composition** | **Family-level composition** | **Feeding trait composition** |
| --- | --- | --- | --- | --- |
| Bare soil (1 km) | 10%* | 4% | 5% | 3% |
| Forest (1 km) | 4% | – | 34% | 46% |
| Humidity | 28% | 23% | 10% | 9% |
| Low vegetation (50 m) | 24% | 12% | 12% | 14% |
| Urban (1 km) | 11% | 15% | – | 3% |
| *H* (50 m) | 5% | 6% | 6% | 2% |

*Values show percentages of the total explained variation (adjusted-R^2^) accounted for by each predictor in each RDA, with land cover heterogeneity represented as ‘*H*’.

**Appendix S15** – Biomass and richness GAMs

**Table S15.1. GAMs with only important predictors.** Generalized additive model relationships between total biomass (g day^-1^) or total species richness (species day^-1^) and the important land use, weather, and climate predictors (see Table 1). The ‘maximum goodness-of-fit’ is the highest adjusted-R^2^ (R^2^_adj_) achieved after forward selection of the individual predictors using maximum likelihood. The subsequent columns then indicate the goodness-of-fit added by each response variable (variables that do not improve the R^2^_adj_ are listed as ‘–‘). Note that the model results shown in Figure 5 include only the variables that improved the R^2^_adj_. Those models use restricted maximum likelihood thus the total R^2^_adj_ can differ slightly from the values shown here.

| **Response** | **Maximum goodness-‌**  **of-fit** (R^2^_adj_) | **Bare soil**  (1 km) | **Forest**  (1 km) | **Low vegetation**  (50 m) | **Urban**  (1 km) | ***H***  (50 m) | **Humidity** |
| --- | --- | --- | --- | --- | --- | --- | --- |
| Biomass | 0.415 | 0.075 | 0.228 | 0.014 | – | – | 0.098 |
| Total richness | 0.476 | 0.088 | – | 0.388 | – | – | – |

**Table S15.2. GAM coefficients.** Coefficients for all smoothed fixed terms from generalized additive models (using restricted maximum likelihood) of the relationship between total biomass (g day^-1^) or total species richness (species day^-1^) and the land use, weather, and climate predictors shown in Table S15.1 that improved the adjusted-R^2^. ‘Edf’ refers to the effective degrees of freedom. Note that differences in the number of samples between biomass and richness occurred because community data was unavailable for several sites (described in *Methods*).

| **Model** | **Term** | **edf** | **F** | ***P*** |
| --- | --- | --- | --- | --- |
| Biomass | Forest:2019 | 1.00 | 8.15 | 0.0051 |
| (*n* = 131) | Forest:2020 | 1.00 | 21.77 | <0.001 |
|  | Low veg:2019 | 1.00 | 4.72 | 0.032 |
|  | Low veg:2020 | 1.00 | 3.97 | 0.049 |
|  | Bare:2019 | 1.00 | 4.18 | 0.043 |
|  | Bare:2020 | 1.00 | 9.94 | 0.0020 |
|  | Humidity:2019 | 4.00 | 2.95 | 0.016 |
|  | Humidity:2020 | 1.83 | 4.79 | 0.0082 |
| Total richness | Low veg:2019 | 2.11 | 6.36 | 0.0020 |
| (*n* = 123) | Low veg:2020 | 4.58 | 8.05 | <0.001 |
|  | Bare:2019 | 1.00 | 1.99 | 0.16 |
|  | Bare:2020 | 2.72 | 6.93 | 0.0024 |
